# Liquid Biopsy in Gastrointestinal Cancers: Circulating Tumor DNA for Molecular Residual Disease Assessment and Early Treatment Monitoring

**DOI:** 10.3390/cancers18061014

**Published:** 2026-03-20

**Authors:** Kamil Safiejko, Marcin Juchimiuk, Jacek Pierko, Maciej Maslyk, Mateusz Mucha, Mariusz Koda, Luiza Konczuga-Koda, Sebastian Radej, Adem Akcakaya, Lukasz Szarpak

**Affiliations:** 1 Colorectal Cancer Unit, Maria Sklodowska-Curie Bialystok Oncology Center, 15-027 Bialystok, Poland; 2 Institute of Biological Sciences, The John Paul II Catholic University of Lublin, 20-708 Lublin, Polandsebastian.radej@kul.pl (S.R.); 3 Department of Pathology, Maria Sklodowska-Curie Bialystok Oncology Center, 15-027 Bialystok, Poland; 4 Department of General Pathomorphology, Medical University of Bialystok, 15-269 Bialystok, Poland; 5 Department of General Surgery, Faculty of Medicine, Bezmialem Vakif University, 34093 İstanbul, Türkiye; 6 Institute of Medical Science, The John Paul II Catholic University of Lublin, 20-708 Lublin, Poland; 7 Henry JN Taub Department of Emergency Medicine, Baylor College of Medicine, Houston, TX 77030, USA

**Keywords:** ctDNA, liquid biopsy, molecular residual disease, mRD, early progression, colorectal cancer, gastric cancer, esophageal cancer, pancreatic cancer, cholangiocarcinoma, hepatocellular carcinoma, clonal hematopoiesis, CHIP

## Abstract

**Background:** Liquid biopsy using circulating tumor DNA (ctDNA) is rapidly reshaping gastrointestinal (GI) oncology. The highest-impact applications are molecular residual disease (mRD) detection after curative-intent therapy and early recognition of progression or resistance during systemic treatment. **Methods:** We performed a structured, clinically oriented narrative synthesis by using explicit search, eligibility, evidence prioritization, and clinical interpretation rules, integrating landmark prospective cohorts, randomized ctDNA-guided strategy trials where available, meta-analyses, key methodological research (e.g., pre-analytics, assay design, and clonal hematopoiesis (CH)/clonal hematopoiesis of indeterminate potential (CHIP)), and selected trial registries. **Results:** In resected colorectal cancer (CRC), postoperative ctDNA positivity is among the strongest known biomarkers of recurrence risk; large prospective studies demonstrate clear separation of disease-free survival (DFS)/overall survival (OS) between mRD+ and mRD− patients. In stage II colon cancer, randomized data (DYNAMIC) show that a ctDNA-guided strategy reduces adjuvant chemotherapy exposure without compromising long-term outcomes. In metastatic CRC, ctDNA supports early response monitoring and resistance tracking; ctDNA-selected anti-EGFR rechallenge provides a model of biomarker-driven actionability (CHRONOS). In gastroesophageal cancers, longitudinal ctDNA dynamics correlate with relapse risk and treatment efficacy, and in esophageal squamous cell carcinoma, ctDNA after neoadjuvant chemoradiotherapy informs residual disease risk and adjuvant stratification. In pancreatic ductal adenocarcinoma and hepatobiliary malignancies, sensitivity is constrained by low shedding and background cell-free DNA (cfDNA), yet ctDNA positivity remains clinically meaningful, and emerging data in resected extrahepatic cholangiocarcinoma (STAMP-linked analyses) show that ctDNA dynamics during adjuvant therapy predict recurrence. **Conclusions:** ctDNA is a clinically validated biomarker for mRD in CRC, whereas in other GI cancers, it remains a promising but methodologically heterogeneous tool whose clinical utility is tumor- and context-dependent. The next phase requires interventional trials demonstrating outcome improvement, harmonized sampling and reporting standards, and rigorous control of confounders (notably CH/CHIP).

## Simple Summary

Circulating tumor DNA (ctDNA) is a blood-based marker that may help doctors detect residual cancer after treatment and identify treatment failure earlier than standard imaging in gastrointestinal cancers. The strongest evidence currently comes from colorectal cancer, where postoperative ctDNA is a powerful predictor of recurrence risk and can guide adjuvant treatment decisions in selected settings. In gastroesophageal, pancreatic, biliary, and liver cancers, ctDNA is promising but less consistent because tumors often shed less DNA into the blood and test performance varies. Positive ctDNA results are usually clinically meaningful, while negative results must be interpreted cautiously. Broader routine use will require standardized testing, careful control of false positives, and trials showing improved patient outcomes.

## 1. Introduction

Gastrointestinal (GI) oncology has made tangible progress, yet relapse after curative-intent therapy and delayed recognition of treatment failure remain common [1,2]. We still often act when disease becomes anatomically visible rather than when it becomes biologically uncontrolled. Serum markers (e.g., CEA, CA19-9, and AFP) remain useful adjuncts, but their sensitivity at minimal tumor burden is limited, and their biology is not tumor-specific enough to safely guide escalation or de-escalation decisions [3,4]. Imaging remains indispensable, but a conventional 8–12-week cadence in metastatic disease is mismatched to clonal selection under treatment pressure, where resistance can emerge weeks before RECIST progression [5,6].

Before any liquid biopsy result can be meaningfully interpreted, GI cancer care remains anchored in a rigorous diagnostic pathway based on high-quality imaging, endoscopy, histopathological confirmation, and multidisciplinary review. This is particularly relevant in high-volume centers, where structured staging, centralization of expertise, and tumor-board decision making are integral to high-quality oncologic care [7]. In this context, ctDNA should be understood as a complementary biomarker that may refine biological risk assessment and treatment monitoring, rather than as a replacement for standard diagnostic work-up or tissue-based evaluation.

Circulating tumor DNA (ctDNA) offers two properties that are clinically consequential [8]. However, the evidentiary maturity and clinical readiness of ctDNA in GI oncology differ substantially by tumor type, assay class, and clinical window. First, it is a short-half-life biomarker: changes can reflect current tumor activity rather than historical burden. Second, it can capture clonal evolution without repeated invasive biopsies, enabling real-time resistance profiling and selection for targeted sequencing strategies [9,10]. For these reasons, the clinically highest-value applications of ctDNA in GI cancers include (i) molecular residual disease (mRD) assessment after curative-intent therapy, (ii) treatment monitoring and early recognition of resistance during systemic therapy, and (iii) selected neoadjuvant or perioperative settings, where serial ctDNA dynamics may provide an early readout of systemic disease control before surgery [11].

Importantly, earlier knowledge does not automatically translate into better outcomes. The evidentiary standard for ctDNA-guided decisions should be interventional: does acting on ctDNA improve overall survival (OS), cure rates, or quality of life, rather than merely advancing the date of relapse recognition? [5,11]. This review therefore focuses on where ctDNA appears the closest to practice-relevant application and whether its use remains better understood as trial-oriented or exploratory.

In day-to-day practice, the harm of delayed recognition is tangible. In the adjuvant setting, we expose large numbers of patients to neurotoxicity, cytopenias, and long-term quality-of-life impairment for a benefit that is substantial in aggregate but uneven at the individual level [12,13]. In metastatic disease, we continue ineffective therapy until progression is unequivocal, at the cost of performance status and eligibility for later lines. ctDNA is attractive because it may allow for earlier, biologically grounded decisions without waiting for an anatomic “threshold” to be crossed [8].

Yet the clinician’s obligation is to resist biomarker enthusiasm that outpaces evidence. A ctDNA test is only as clinically valuable as the action it triggers. For this reason, we deliberately distinguish between ctDNA as a prognostic classifier and ctDNA as a decision tool. The former is supported in several GI settings to varying degrees; the latter requires strategy trials showing that ctDNA-guided escalation or switching improves outcomes rather than simply shifting the time of detection. Throughout this review, we treat ctDNA as a tumor- and context-dependent biomarker rather than a uniform analyte, because the molecular target, shedding behavior, and clinical meaning of plasma detection differ substantially across GI malignancies.

## 2. Materials and Methods

We prepared this review as a clinically driven, structured narrative synthesis. The intent was not to reproduce an exhaustive PRISMA workflow but to answer the questions that actually determine bedside decisions in GI oncology: When does ctDNA meaningfully change post-curative risk assessment? When can it inform earlier recognition of treatment failure or resistance? What technical and biological conditions must be met before a negative or positive result can be trusted? We used current consensus guidance on ctDNA reporting and interpretation (particularly around pre-analytics, analytical sensitivity, and clonal hematopoiesis) to define the minimum evidentiary and methodological standards required for inclusion and interpretation [14,15,16].

### 2.1. Data Sources, Search Strategy, and Currency of Evidence

Primary searches were performed in PubMed/MEDLINE, supplemented by targeted backward citation tracking from pivotal primary studies, contemporary reviews, and guideline statements. Search strings combined disease-agnostic ctDNA terms (“circulating tumor DNA”, “ctDNA”, “cell-free DNA”) with clinical use-case terms (“molecular residual disease”, “minimal residual disease”, “MRD”, “surveillance”, “monitoring”, “kinetics”, “resistance”, “rechallenge”) and tumor-specific terms (“colorectal”, “gastric”, “gastroesophageal”, “esophageal”, “pancreatic”, “cholangiocarcinoma”, “biliary tract”, “hepatocellular carcinoma”). Because assay validity and interpretability in mRD depend disproportionately on the analytical method, we also used methodological terms (“error suppression”, “unique molecular identifiers”, “duplex sequencing”, “clonal hematopoiesis”) and anchored key technical claims in seminal methodological papers [17,18,19]. PubMed/MEDLINE was searched from database inception to 6 February 2026. The backward citation tracking of pivotal studies, reviews, and guideline statements was last performed on 6 February 2026. The full reproducible PubMed/MEDLINE search strings are provided in Supplementary Table S1.

### 2.2. Inclusion/Exclusion Criteria and Evidence Prioritization

Given the narrative design, we used explicit eligibility criteria to reduce selection bias while preserving a practice-oriented focus.

Included were English-language, peer-reviewed original studies and meta-analyses with clearly described search methods, explicit eligibility criteria, study-level assay characterization, and clinically relevant endpoints that reported at least one of the following: (i) time-to-event endpoints (disease-free survival (DFS)/overall survival (OS)/progression-free survival (PFS)) in relation to ctDNA/mRD status or dynamics, (ii) clearly defined sampling windows paired with interpretable assay descriptions (assay class, signal type, and analytical sensitivity), or (iii) clinically actionable resistance profiling linked to treatment selection or sequencing. We included both tumor-informed and tumor-agnostic approaches and both mutation-based and methylation/fragmentomic assays but treated them as non-interchangeable unless the clinical window and assay performance were directly comparable [17,20,21].

Excluded were single-patient case reports; small case series lacking the minimum methodological transparency required for interpretation, defined as the reporting of assay class, signal type, sampling window, and either analytical sensitivity metrics (e.g., LOD/LoQ) or a validated error-suppression and/or CH/CHIP mitigation approach; studies without time-to-event or clinically interpretable response endpoints; and reports in which mutation-only tumor-agnostic results could not reasonably be distinguished from clonal hematopoiesis (CH)/clonal hematopoiesis of indeterminate potential (CHIP) risk because CH mitigation was absent or unclear.

For this review, interpretability was defined pragmatically as the minimum level of methodological reporting needed to judge whether a ctDNA result could function as a credible rule-in or rule-out signal in the stated clinical setting.

To keep the synthesis aligned with clinical decision making, we applied a prespecified hierarchy of evidence:

1. Randomized ctDNA-guided strategy trials with mature follow-up when available [11,22];
2. Large prospective cohorts/registries assessing post-treatment mRD and longitudinal trajectories with DFS/OS endpoints [23,24,25];
3. Prospective longitudinal monitoring studies relating early ctDNA kinetics and/or resistance profiles to response and outcomes in metastatic disease [26,27,28];
4. Methodological studies essential to interpretation (pre-analytics, error suppression, detection thresholds, and CH/CHIP confounding) [14,17,19];
5. Guidelines, consensus statements, and whitepapers informing implementation constraints and reporting expectations [14,15,16].

For the purposes of evidence prioritization, “large prospective cohorts” were defined pragmatically as prospective multicenter, registry-based, or otherwise methodologically mature cohorts with prespecified sampling windows, clinically interpretable endpoints, and sample sizes generally ≥100 patients; in rarer disease settings where such datasets were unavailable, the largest available prospective studies were retained but interpreted with proportionately greater caution.

Because ctDNA detectability is strongly shaped by tumor shedding biology and disease compartment, we emphasized tumor- and scenario-specific interpretation rather than applying uniform thresholds across GI malignancies [17,20,21].

Study selection was performed in a staged manner. Titles and abstracts were first screened for relevance to the prespecified clinical questions and for minimum methodological interpretability. Full texts of potentially eligible records were then assessed against the criteria above. When multiple publications arose from the same cohort, registry, or platform program, we preferentially cited the most contemporary and clinically informative report while retaining earlier publications only when needed to clarify assay design, sampling schema, or the evolution of the evidence base. Trial-registry entries were used to contextualize ongoing interventional paradigms and evidence gaps but were not treated as equivalent to peer-reviewed outcome data. The search and citation-tracking process identified 740 records in total, where 195 articles underwent full-text assessment, and 73 studies were retained for the final structured narrative synthesis.

### 2.3. Trial Registries and Active Interventional Paradigms

To ensure contemporaneity and avoid presenting the field as static, we cross-referenced major trial registries (e.g., ClinicalTrials.gov and relevant national registries) to identify active ctDNA-guided platform programs and ongoing escalation/de-escalation paradigms in resected and metastatic GI cancers. Registry entries were used to contextualize evidence gaps, clarify which questions are being tested prospectively, and prevent extrapolation beyond what interventional evidence currently supports [29,30,31].

### 2.4. Data Abstraction and Clinical Synthesis

For each included study, we abstracted tumor type, clinical setting (post-curative mRD, on-treatment monitoring, and surveillance), assay class (tumor-informed vs. tumor-agnostic; mutation-based vs. methylation/fragmentomics), sampling schedule, endpoint definitions, and the direction and magnitude of association with outcomes. The synthesis was organized a priori into four clinically relevant domains: (i) biological and technical interpretability, (ii) post-curative molecular residual disease, (iii) early progression/resistance during systemic therapy, and (iv) implementation, pitfalls, and future directions. Within each domain, evidence was weighted according to assay validity, sampling window, endpoint relevance, and direct clinical actionability. We intentionally synthesized the literature clinically rather than statistically. Accordingly, the aim of this review was not to generate pooled effect estimates across heterogeneous assays and clinical windows but to derive defensible interpretive principles, including rule-in versus rule-out value, asymmetric error costs, and clinically meaningful ctDNA trajectories (persistent mRD−, mRD+ clearance, persistent mRD+, and conversion from mRD− to mRD+). When results diverged across studies, differences in timing, assay class, and shedding biology were treated as first-order explanations rather than noise [14,15,19]. Conclusions were framed conservatively whenever evidence was observational, indirect, or not yet linked to a validated downstream management strategy. Where this manuscript proposes practical algorithms, monitoring schedules, or minimum-action pathways, these should be understood as author-derived clinical interpretation frameworks informed by the cited literature, not as formal guideline recommendations or substitutes for trial-based evidence. To provide quantitative anchoring without implying cross-study comparability that the heterogeneous literature does not support, representative hazard ratios, survival estimates, and test-performance parameters from pivotal studies and meta-analyses are summarized in Supplementary Table S2. Within each tumor-specific section, we first summarize the direct empirical evidence (randomized trials, prospective cohorts, and meta-analyses where appropriate) and then, where clinically useful, present clearly signposted author-derived interpretive proposals intended to operationalize the literature rather than replace trial-based evidence.

### 2.5. Anchoring ctDNA Within Contemporary Standards of Care

We deliberately anchored ctDNA use cases within modern treatment standards (perioperative therapy in upper-GI cancers, adjuvant strategies in colorectal cancer (CRC) and pancreatic ductal adenocarcinoma (PDAC), and contemporary systemic regimens in hepatocellular carcinoma (HCC) and biliary tract cancer (BTC)) because the clinical value of ctDNA depends on whether an actionable alternative exists at the time the result becomes available. This prevents technology-first conclusions and keeps recommendations tethered to what can realistically be offered in practice [32,33,34].

### 2.6. Transparency, Limitations, and Safeguards Against Over-Claiming

This review was designed as a structured, clinically oriented narrative synthesis rather than as a formal systematic review or meta-analysis. Accordingly, the purpose of Section 2 is to make evidence identification, prioritization, and interpretation transparent, not to imply PRISMA-style exhaustiveness.

As a narrative review, we did not undertake a formal instrument-based risk-of-bias appraisal for each included study. Instead, we used a clinically oriented evidence prioritization framework that gave greater weight to randomized strategy trials, prospective cohorts, and studies with sufficient methodological reporting to support interpretation. We also treated assay heterogeneity, sampling window, and shedding biology as central determinants of how confidently individual findings could be applied clinically. In keeping with this design, statements implying clinical action were framed cautiously and in proportion to the maturity of the underlying evidence, particularly where data were observational or where biological constraints limited rule-out value [14,15,16].

## 3. Biological and Technical Foundations for Clinical Use

From a clinician’s perspective, ctDNA results must be interpretable, reproducible, and reportable in a way that supports decision making [35,36]. At minimum, reports should specify assay class (tumor-informed vs. tumor-agnostic), signal type (mutations vs. methylation/fragmentomics), limit of detection/quantification (LOD/LoQ), plasma volume and cell-free (cfDNA) yield, and the error-suppression strategy (e.g., unique molecular identifiers, duplex sequencing, and digital error suppression) [35]. Without these parameters, a negative result may be over-interpreted, and cross-study comparisons become unreliable [36]. To support cross-study comparability and transparent interpretation, a minimum reporting framework and curated additional evidence map are provided in Supplementary Table S3.

Tumor shedding biology is the dominant determinant of ctDNA detectability [37]. Detectability reflects not only tumor burden but also vascularity, metastatic distribution, cellular turnover, anatomic compartments (peritoneal-dominant disease), histology (mucinous tumors), and inflammatory background increasing cfDNA [38]. This explains why a negative result is often more reassuring in CRC than in pancreatic ductal adenocarcinoma (PDAC) or HCC and why longitudinal trends frequently outperform single timepoints.

Assay classes matter because they define sensitivity in mRD. Tumor-informed assays track multiple patient-specific variants and generally maximize mRD sensitivity [39]. Tumor-agnostic mutation panels are faster and tissue-free, but their negative predictive value in mRD is constrained by breadth and by CH/CHIP confounding. Non-mutation signals (methylation and fragmentomics) may improve detection at ultra-low tumor fractions but must be validated in the specific clinical setting (e.g., post-resection PDAC) rather than extrapolated from screening cohorts [39,40]. Table 1 provides a practical assay selection cheat-sheet (tumor-informed vs. tumor-agnostic and tissue-free signatures), including how to phrase “negative” results safely across clinical scenarios.

Importantly, ctDNA in gastrointestinal oncology should not be viewed as a single universal biomarker. Rather, the molecular substrate interrogated in plasma varies across tumor types and clinical scenarios. In colorectal and pancreatic cancers, mutation-based approaches frequently rely on recurrent driver alterations and patient-specific variants identified from the primary tumor. In biliary tract cancers, ctDNA may also capture therapeutically actionable genomic alterations directly in plasma, whereas in hepatocellular carcinoma, methylation- and fragmentomics-based approaches may be particularly informative in high-background-cfDNA settings. Accordingly, assay performance and clinical interpretation are tumor-specific rather than interchangeable. The significance of a positive or negative ctDNA result depends not only on analytical sensitivity but also on the molecular target being tracked, the method by which it was identified, and the extent to which the underlying tumor biology supports reliable plasma shedding. These tumor-specific distinctions are synthesized in Table 2 as a biologically and clinically oriented interpretive framework; the landmark empirical studies supporting these disease-specific sections are summarized separately in Supplementary Table S2.

The principal confounder is clonal hematopoiesis (CH/CHIP). Hematopoietic clones contribute somatic variants to cfDNA and can mimic tumor alterations, particularly in tumor-agnostic single-nucleotide variant (SNV) assays [14]. mRD workflows should incorporate matched white blood cell sequencing or robust CH filtering and reporting; otherwise, clinically meaningful false positives may drive inappropriate escalation [41]. A practical checklist for recognizing and mitigating CH/CHIP-driven false positives (with minimum lab/bioinformatics safeguards and safest clinical handling) is provided in Supplementary Table S4.

Timing is a clinical variable, not a technical footnote. For ctDNA results to be comparable across studies and interpretable in practice, sampling windows should be prespecified rather than opportunistic. When feasible, a baseline sample should be obtained before surgery or before systemic therapy, as this establishes whether ctDNA is measurable in that patient and anchors interpretation of subsequent negative results. In the postoperative setting, sampling performed too early may be confounded by the postoperative rise in cfDNA and dilution of tumor fraction; therefore, molecular residual disease assessment is best performed after this immediate postoperative phase has subsided and within a predefined window that still preserves relevance for adjuvant decision making. End-of-treatment assessment should likewise be performed within a standardized post-treatment window rather than at arbitrary timepoints. In metastatic disease, early on-treatment kinetics are most informative when blood is drawn at fixed intervals from treatment initiation, typically at baseline and again within the first 2–4 weeks, provided that baseline ctDNA is measurable and that the same assay is used longitudinally. Thus, there is no single universal “best” time for liquid biopsy across all GI cancers; rather, clinically meaningful standardization depends on scenario-specific, predefined sampling windows.

Pre-analytics are particularly critical in mRD because tumor fraction can be near the detection limit. Delayed plasma separation, hemolysis, or inadequate plasma volume can shift a true low positive into a false negative [41,42]. Centers should therefore treat the “ctDNA pathway” as a clinical service with quality metrics (time-to-spin, plasma volume targets, and sample rejection criteria), analogous to transfusion medicine or molecular pathology workflows. Minimum pre-analytical and reporting QC standards for ctDNA (including rejection rules and “if–then” reporting guardrails) are provided in Supplementary Table S5.

An underappreciated point is that ctDNA is a systemic plasma marker; it is less sensitive for compartmentalized disease such as peritoneal-only metastases or certain mucinous phenotypes [43]. In these scenarios, discordance between symptoms, imaging, and ctDNA should not be resolved in favor of ctDNA. Instead, it should trigger a deliberate reconsideration of disease compartment and imaging modality (e.g., liver MRI or diffusion-weighted sequences; consideration of PET/CT in selected contexts) [44].

Finally, when ctDNA is used for resistance profiling, clinicians should interpret “absence of a resistance variant” as conditional on assay breadth and sensitivity. For example, a negative RAS panel in plasma is not equivalent to “no resistant clone exists”; it means that no clone is present above the assay’s detection threshold in plasma at that time. This distinction is critical when decisions include re-exposure to targeted agents [14,18]. Methodological foundations, error-suppression approaches, and clonal hematopoiesis confounding are supported by seminal technical and clinical studies [14,18,19].

## 4. Clinical Interpretation: mRD Positivity Is Actionable; Negativity Is Contextual

**Figure 1:**
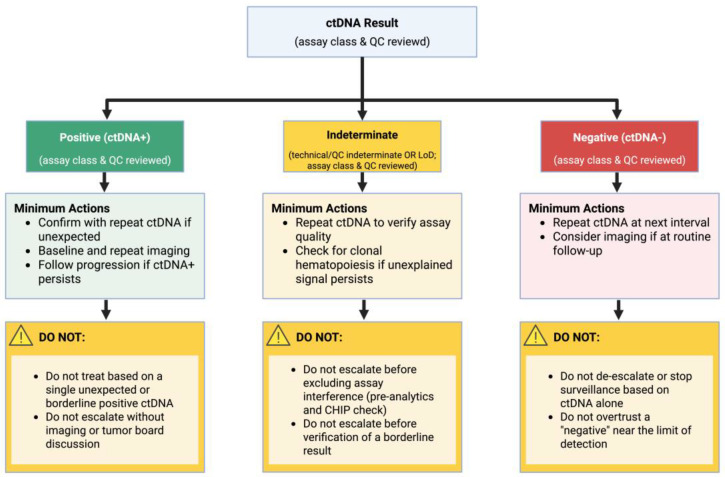
Clinical interpretation of ctDNA results with guardrails for responsible use. Flowchart summarizing minimum actions for ctDNA-positive, ctDNA-negative, and indeterminate (technical/QC or near-LoD) results, including confirmatory repeat testing, imaging escalation, pre-analytics/QC review, and CH/CHIP consideration. The “DO NOT” boxes highlight common unsafe actions (e.g., treatment escalation or surveillance de-escalation based on a single result without verification and multidisciplinary review). Author-derived interpretive framework based on the cited literature. Created in BioRender. Szarpak, L. (2026) https://BioRender.com/81407er(accessed on 18 March 2026).

mRD should be interpreted through the lens of asymmetric error costs. A positive post-curative ctDNA result is usually a strong “rule-in” signal for residual disease, whereas the “rule-out” value of a negative result is tumor- and assay-dependent [13]. In high-shedding contexts (CRC), mRD- is relatively reassuring, particularly when baseline ctDNA was measurable and the assay has high sensitivity [45]. In low-shedding contexts (e.g., PDAC, HCC, and peritoneal-dominant disease), mRD- often means no detectable plasma signal with this assay at that time—not absence of disease [11,14,46]. A pragmatic interpretation algorithm with minimum actions and “do not” guardrails is shown in Figure 1. Biology-driven scenarios where a negative ctDNA result should not reassure (and minimum-harm mitigation strategies) are summarized in Supplementary Table S8.

The practical interpretation framework below is intended to operationalize the literature for bedside decision making; where interventional evidence is lacking, it reflects cautious author synthesis rather than validated trial-derived management rules.

A pragmatic two-question framework helps prevent over-interpretation: (1) Was ctDNA measurable at baseline (patient-specific shedding)? (2) What clinical action will follow a positive or negative result? If no action follows, testing risks becoming earlier anxiety rather than decision support [47]. Where action is possible, mRD+ should trigger a predefined pathway: confirm persistence when borderline, review sample quality and CH/CHIP control, escalate imaging thoughtfully (e.g., liver MRI when appropriate), and prioritize trial enrollment for mRD-guided intensification. Conversely, de-escalation based solely on a single mRD- in high-stakes settings (e.g., stage III colon cancer) should remain trial-restricted [47,48].

A useful clinical habit is to document, at the time of ordering, the intended downstream actions for both outcomes. For example: “If mRD+ pre-adjuvant, recommend ACT and consider trial; if mRD-, follow standard risk-based approach”. This prevents retrospective rationalization and keeps ctDNA aligned with decision quality [49,50].

Another practical principle is that mRD status should rarely be acted on from a single borderline value. If the result is low-level positive, repeat sampling within a short, predefined interval can help distinguish transient noise from persistent signal [50,51,52]. Similarly, for unexpected mRD positivity, the clinician should explicitly consider CH/CHIP and request clarification of how the laboratory mitigated it.

Where mRD is used in surveillance, the key is to avoid “unstructured escalation”. A coherent pathway might include: repeat ctDNA to confirm a rising trend, targeted imaging optimized for the most likely relapse sites (e.g., liver MRI in CRC), multidisciplinary review for oligometastatic salvage, and trial referral if imaging remains negative. Without such a pathway, surveillance risks earlier anxiety without a survival dividend [14,47]. Suggested ctDNA monitoring schedules, rules for trend interpretation, and defensible clinical responses across key GI settings are summarized in Supplementary Table S7.

## 5. Colorectal Cancer: mRD and Early Progression

CRC is the reference model for ctDNA mRD because tumor shedding is relatively high, endpoints are well-defined, and the postoperative decision about adjuvant chemotherapy (ACT) is tangible [53]. Classical pathologic risk factors estimate probability; ctDNA mRD positivity is biological evidence of residual malignant material and therefore has the potential to become predictive of treatment benefit—especially in the adjuvant window, when eradication of microscopic disease remains plausible [54,55].

Large prospective programs consistently show that postoperative ctDNA positivity identifies a group with markedly increased recurrence risk, with clear separation of DFS and OS between mRD+ and mRD− patients. Clinically, this supports treating mRD+ as molecularly detectable systemic disease even when imaging is negative [54]. Importantly, mRD is most informative when interpreted longitudinally. Four clinically useful trajectories recur in practice: sustained mRD−; mRD+ with clearance on ACT; persistent mRD+ despite ACT; and conversion from mRD− to mRD+ during surveillance [53,54]. The latter two trajectories should trigger predefined escalation pathways—confirmatory repeat testing, high-quality targeted imaging (often liver MRI), and consideration of trial if imaging remains negative—rather than passive observation [54,56]. Table 3 summarizes practical ctDNA-defined mRD trajectories after curative-intent CRC resection and suggested minimum-action pathways. Representative quantitative anchors for postoperative risk stratification and ctDNA-guided adjuvant strategy data in CRC are summarized in Supplementary Table S2.

Stage II colon cancer is where ctDNA has the strongest strategy-level evidence [54]. DYNAMIC tested a ctDNA-guided management approach in which ACT was largely reserved for postoperative ctDNA-positive patients, whereas standard care used clinicopathologic features to guide ACT use [48,54]. Mature 5-year outcomes demonstrated non-inferior oncologic results with substantially reduced chemotherapy exposure, supporting ctDNA-guided risk-adapted management in stage II disease [52,54,56]. Practically, postoperative mRD+ strengthens the indication for ACT and justifies the closer monitoring of clearance, while postoperative mRD− supports observation—particularly when conventional high-risk features are absent or marginal and toxicity concerns are prominent [54].

In stage III colon cancer, de-escalation remains a trial question because ACT benefit is established at the population level and the acceptable false-negative rate is low [45,54]. For now, mRD− should be viewed as supportive context for tailoring regimen intensity and duration (within validated clinical risk groups and tolerability constraints), not as a stand-alone reason to omit therapy outside trials. Conversely, mRD+ identifies very high-risk biology that is well-suited to intensification studies and closer follow-up [54,57,58].

Serial ctDNA surveillance frequently detects molecular relapse earlier than radiology, but the clinical value depends on actionability [59]. Earlier detection matters when it enables metastasis-directed therapy for oligorecurrence, accelerates trial enrollment, or allows for systemic treatment in a minimal-disease window. Without a predefined intervention plan, surveillance risks generating lead time without outcome benefit and can increase anxiety and low-value testing [40].

In metastatic CRC, the most mature “actionable” role of ctDNA in GI oncology is resistance tracking under anti-EGFR therapy [60]. Emergence of RAS/BRAF alterations and EGFR extracellular domain variants in ctDNA can precede RECIST progression [61]. After anti-EGFR withdrawal, resistant clones may decay, creating a biological window for rechallenge. CHRONOS operationalized this concept by selecting patients for panitumumab rechallenge based on ctDNA profiles, translating resistance biology into rational sequencing [61]. In routine practice, ctDNA should not replace imaging, but it can justify earlier reassessment, help explain discordant radiology, and guide rechallenge selection when resistance variants are absent [60]. Table 4 summarizes ctDNA-based decision triggers for anti-EGFR-treated mCRC, including resistance monitoring and rechallenge guardrails.

For tumor-board decision making, postoperative mRD+ reframes the discussion toward two pragmatic questions: whether an evidence-supported intensification strategy (or trial) is available and whether relapse could be localized early enough for metastasis-directed therapy [54]. For mRD− patients, the question is not “cured versus not cured,” but whether standard ACT intensity and duration can be reasonably tailored based on combined clinicopathologic risk, expected benefit, and treatment tolerance [44,54].

ctDNA-guided management is also not limited to the “yes/no chemotherapy” decision. End-of-treatment and early post-ACT sampling can identify patients with persistent molecular disease—a group in whom conventional follow-up often detects relapse late. In practice, persistent mRD after ACT should trigger a heightened diagnostic posture and consideration of escalation or novel-agent trials rather than routine-interval surveillance [53].

The anti-EGFR rechallenge paradigm also illustrates a broader biological principle: resistant clones emerge under drug pressure and decay once pressure is removed. ctDNA allows clinicians to measure this decay rather than infer it, converting a previously empirical rechallenge strategy into a biomarker-informed one with clearer selection logic [60].

Several CRC contexts deserve special handling. In rectal cancer treated with total neoadjuvant therapy, ctDNA can complement response assessment by informing whether systemic control has been achieved even when local response appears favorable [62]. In MSI-high disease, ctDNA may support longitudinal monitoring and potentially detect molecular relapse, but its role in immunotherapy monitoring requires careful validation and should be interpreted alongside clinical and radiologic data [60].

Two practical pitfalls recur in CRC implementation. One is using a resistance-focused plasma panel (optimized for common actionable variants) as a surrogate mRD assay, which can yield false-negative reassurance because it is not designed for ultra-low tumor fractions. The other is acting on a single isolated positive result without CHIP/CH control or confirmatory testing. Both are preventable with governance: align assay choice to the clinical indication, and adopt a repeat-testing policy for borderline or unexpected results [54,63].

Even in CRC, ctDNA does not replace clinicopathologic reasoning; it reorganizes it. A coherent approach is hierarchical: use mRD to define biological risk, then use classic features to refine clinical risk and tolerability trade-offs. For example, a stage II patient with T4 disease but mRD− may reasonably avoid ACT if toxicity risk is high, whereas an mRD+ patient without conventional high-risk features should still be managed as high risk. This reframes traditional risk models rather than abolishing them [14,18,19].

CRC evidence spans prospective mRD cohorts, randomized ctDNA-guided strategy trials, established adjuvant standards, resistance biology, and active platform-trial ecosystems [22,30].

## 6. Gastroesophageal and Esophageal Cancers

Gastroesophageal cancers (gastric/GEJ and esophageal) have a distinct ctDNA biology in which detectability is more variable than in CRC and negative results are therefore less reassuring. In this disease group, one of the most practical near-term roles of ctDNA may lie not only after surgery but also during neoadjuvant or perioperative therapy, where longitudinal dynamics can complement radiologic and pathologic assessment of systemic control. Even so, a positive ctDNA result after curative-intent therapy is consistently a high-risk signal and should be treated as biologically meaningful rather than incidental [64,65]. Representative quantitative data for non-CRC GI settings are likewise summarized in Supplementary Table S2, with the explicit caveat that assay class, sampling windows, and endpoints differ substantially across tumor types.

In perioperative gastric and GEJ cancer, modern regimens have improved outcomes, yet a subset of patients relapses early despite apparently adequate local control. Longitudinal ctDNA assessment during neoadjuvant therapy and early after surgery can function as a pragmatic test of systemic control: persistent detectability suggests biologically active systemic disease despite anatomic resectability. Prospective biomarker frameworks such as PLAGAST illustrate how ctDNA dynamics across treatment can be linked to response and relapse risk. Clinically, postoperative ctDNA positivity should support intensified surveillance, a lower threshold for targeted imaging (particularly liver and peritoneum), and consideration of interventional trials when available [65,66].

In esophageal squamous cell carcinoma, the post-nCRT interval is characterized by uncertainty because imaging and endoscopic assessment have limited sensitivity for microscopic residual disease [67]. Emerging data suggest that ctDNA positivity after nCRT can identify residual disease risk and may help guide adjuvant stratification and surveillance intensity, especially now that adjuvant immunotherapy is established for selected high-risk patients [68]. In this setting, ctDNA should be interpreted alongside pathology, imaging, and endoscopic findings; neither positivity nor negativity should be treated in isolation [67,69].

In advanced gastroesophageal disease, clinical reserves can deteriorate quickly. When baseline ctDNA is measurable, early kinetics at 2–4 weeks may help identify biological non-responders before radiographic progression becomes obvious, supporting earlier imaging or earlier reassessment of strategy. However, the evidence base remains largely correlational, and strategy trials are still needed before ctDNA-guided switching can be considered routine [70].

In the perioperative clinic, persistent ctDNA after neoadjuvant therapy should generally be interpreted as suboptimal systemic control rather than a technical failure, particularly when baseline ctDNA was detectable. This can justify closer surveillance and a lower threshold to investigate nonspecific symptoms that might otherwise be attributed to post-treatment effects [71]. Conversely, ctDNA clearance during neoadjuvant therapy is biologically reassuring but does not yet justify de-escalation of established perioperative standards outside clinical trials [70].

The post-nCRT “organ preservation” context in esophageal cancer also illustrates the danger of false reassurance. A negative ctDNA result should not be used as a stand-alone justification for nonoperative management unless embedded in validated protocols that integrate endoscopy, biopsies, and high-quality imaging [69]. The most defensible current role is risk stratification for surveillance intensity and trial enrollment for adjuvant intensification strategies.

In frail patients with advanced disease, an early ctDNA non-response signal can ethically support earlier imaging or a shift toward supportive-focused care, potentially preserving quality of life. Using ctDNA to avoid futile toxicity is as legitimate as using it to intensify therapy, provided the pathway is explicit and evidence-aware [26,71].

Upper-GI context and ctDNA evidence informing these points include perioperative standards, nCRT/adjuvant immunotherapy, and emerging ctDNA kinetics/MRD cohorts in gastroesophageal and esophageal cancers [26,71,72].

## 7. Pancreatic Ductal Adenocarcinoma

In PDAC, mRD assessment is clinically attractive because relapse rates remain high after resection even with modern perioperative therapy; however, its broader applicability is constrained by low plasma shedding and substantial inflammatory cfDNA background [73]. However, ctDNA detection is biologically constrained: PDAC often sheds little tumor DNA into plasma and is accompanied by substantial inflammatory cfDNA background [74]. This creates an important interpretive asymmetry—postoperative mRD positivity is a strong alarm signal for micrometastatic disease, whereas postoperative mRD negativity is frequently non-informative unless baseline ctDNA was measurable and then clears [74,75].

Because no single biomarker is sufficient in PDAC, one pragmatic near-term approach is a composite model that integrates ctDNA as a rule-in signal with CA19-9 trends and high-quality imaging [76]. When postoperative ctDNA positivity is concordant with a rising CA19-9, the likelihood of early systemic relapse is high, supporting maximization of systemic therapy within tolerance, shorter imaging intervals, and early referral for clinical trials [77,78]. When results are discordant, the safer response is repeat sampling and a review of pre-analytics, assay class, and imaging quality rather than automatic escalation based on one signal [75].

Perioperative dynamics and “clearance” are conceptually attractive: ctDNA clearance during neoadjuvant therapy or early after surgery would suggest improved systemic control, whereas persistent detectability suggests resistant or extensive micrometastatic disease. In practice, low baseline detectability limits how broadly this can be applied [73]. For centers implementing ctDNA in PDAC, obtaining baseline plasma before neoadjuvant therapy is advisable even if the primary clinical question is postoperative mRD, because baseline detectability anchors interpretation of any later negative result [73,77].

In advanced PDAC, ctDNA monitoring is most useful when baseline ctDNA is measurable [79]. In that setting, a lack of early decline can support earlier imaging or strategy reassessment, particularly in patients with borderline performance status where time lost on ineffective therapy matters. In patients with undetectable baseline ctDNA, ctDNA monitoring cannot substitute for careful clinical assessment, CA19-9 (when informative), and serial imaging [80].

Clinically, the reality is that postoperative PDAC relapse is common and often rapid; when mRD is positive after surgery, it should be treated as a high-risk state that triggers proactive action—systemic therapy optimization, nutrition and performance support, and early trial screening [81]. The aim is to avoid the common scenario in which relapse becomes radiographically obvious only after the patient has lost fitness for effective systemic treatment [73].

Assay choice also matters. KRAS-focused ddPCR can be attractive for speed and sensitivity when a tracked KRAS mutation is known, but it misses non-KRAS biology and cannot capture heterogeneous subclones. Tumor-informed multi-variant panels may improve sensitivity by aggregating signal, but they remain limited by low shedding. A pragmatic approach is to match the assay to the clinical question: rapid on-treatment kinetics when a trackable KRAS mutation exists versus broader mRD assessment when tissue is available and turnaround time is acceptable [82].

For early progression monitoring in metastatic PDAC, ctDNA kinetics can be informative when the baseline is positive; for example, lack of decline at 2–4 weeks can justify earlier imaging and earlier transition to the next line. However, premature switching based on a single noisy value should be avoided; a trend-based approach combined with symptoms and CA19-9 is safer and better aligned with PDAC biology [83,84].

A further complication is the “compartment problem”: some patients relapse with peritoneal or local–regional disease that may be under-represented in plasma. In these cases, ctDNA negativity should not override symptoms, rising CA19-9, or suspicious imaging. If ctDNA remains repeatedly negative despite high clinical suspicion, clinicians should optimize imaging (e.g., high-quality pancreatic protocol CT/MRI) and consider alternative sampling approaches in research contexts [76].

Looking ahead, the most plausible near-term decision impact of mRD in PDAC is not omission of therapy, but prioritization—identifying who needs the earliest and most intensive systemic therapy, supportive optimization, and trial enrollment [75,76]. Another potential decision node is end-of-treatment assessment: persistent ctDNA positivity after perioperative therapy could enrich patients for maintenance or intensification trials, analogous to mRD-guided escalation strategies in CRC [75]. PDAC considerations here are anchored in contemporary systemic and perioperative standards and ctDNA MRD/monitoring cohorts and meta-analyses [76,81,85]. Representative quantitative anchors and landmark PDAC studies are summarized in Supplementary Tables S2 and S9.

## 8. Biliary Tract Cancer

Biliary tract cancer (BTC) represents a clinically important but still evolving use case for ctDNA, because relapse risk is high after resection, tumor tissue is often limited, and treatment selection in advanced disease increasingly depends on actionable molecular alterations. ctDNA may therefore serve two complementary roles: prognostication via mRD assessment and molecular profiling to identify targetable drivers and emerging resistance [86].

In the post-resection setting, available analyses in resected extrahepatic cholangiocarcinoma indicate that ctDNA status and on-treatment dynamics during adjuvant therapy stratify recurrence risk and separate risk trajectories [87]. Clinically, persistent ctDNA positivity during adjuvant therapy should be interpreted as a high-risk signal supporting intensified surveillance and early consideration of clinical trials when available, rather than waiting for radiographic relapse, which is often detected at a more advanced stage [87,88].

In advanced BTC, ctDNA is frequently the most feasible route to comprehensive genomic profiling when tissue is scarce or unsafe to obtain, including detection of alterations with direct therapeutic relevance such as IDH1 mutations and FGFR2 fusions [89]. Serial ctDNA can also track the emergence of resistance under targeted therapies [64]. This integration is particularly valuable when biliary obstruction, stenting, cholangitis, and inflammation confound serum markers and can blur radiographic interpretation, making a blood-based molecular readout an informative adjunct to imaging and clinical status [90,91,92].

The adjuvant problem in BTC mirrors CRC: we treat broad populations because we cannot reliably identify patients who are already molecularly cured. ctDNA offers a direct biological signal, and persistent positivity during adjuvant therapy is especially concerning. When this occurs, earlier trial discussions are reasonable because waiting for imaging-defined relapse may mean that the disease is already clinically significant at detection [64,89]. A study-level summary of the key post-resection and profiling datasets in BTC is provided in Supplementary Table S9.

In routine care, plasma profiling is often the most practical way to identify actionable alterations and is not simply a matter of convenience: timely recognition of IDH1 mutations or FGFR2 fusions can change sequencing and expand options beyond chemotherapy. Serial ctDNA can then be used to follow resistance evolution, enabling more rational sequencing than reactive switching after overt progression. Evidence informing these points includes systemic and targeted-therapy data in BTC and ctDNA prognostic/profiling cohorts, including post-resection dynamics [93,94,95].

## 9. Hepatocellular Carcinoma

In HCC, ctDNA interpretation is complicated by cirrhosis, necroinflammation, and high background cfDNA, which reduce tumor fraction and can limit mutation-only approaches [27,96]. This strengthens the rationale for incorporating methylation and fragmentomic signals in high-background settings, but these modalities require setting-specific validation [64,97].

After resection or ablation, available evidence supports an association between postoperative ctDNA positivity and recurrence risk, but methodological heterogeneity across studies is substantial (assays, timing, and thresholds) [27]. In practice, postoperative ctDNA positivity may be interpreted as a high-risk signal that can support closer surveillance in selected patients, particularly when liver function is preserved and repeat curative interventions remain feasible. By contrast, ctDNA negativity should not justify de-escalation of surveillance because low shedding and elevated background cfDNA limit rule-out value [64,71].

Representative quantitative data and key HCC studies are summarized in Supplementary Tables S2 and S9, with explicit attention to assay heterogeneity and background-cfDNA constraints.

Because HCC care is shaped by competing risks (tumor relapse versus liver decompensation) and because liver function determines eligibility for many therapies, ad hoc testing can create “knowledge without consequence” or trigger unhelpful downstream actions [41]. Implementation is best pursued within protocolized pathways that prespecify sampling schedules and management responses (e.g., earlier MRI, transplant pathway review, and adjuvant trial screening), so results translate into consistent, actionable decisions [27,98].

From the hepatology–oncology interface, mRD testing should be aligned with realistic intervention options. If earlier relapse detection will not change management—such as in decompensated cirrhosis without a transplant pathway—routine testing may not be justified [61,99].

Conversely, among patients eligible for transplant evaluation or repeat local therapy, earlier molecular signals could plausibly be valuable, but this is also where prospective trials are most needed [64].

HCC considerations should be interpreted in the context of modern systemic therapy standards, guideline frameworks, and the emerging (still heterogeneous) ctDNA MRD evidence base.

## 10. Early Progression and Resistance: From “Knowing Earlier” to “Treating Better”

Early ctDNA kinetics often correlate with outcomes across solid tumors, but GI oncology requires a higher evidentiary bar: earlier detection is clinically meaningful only if it changes management in a way that improves overall survival, cure rates, or quality of life [27,96]. This distinction—correlation versus strategy—should guide implementation.

ctDNA can precede RECIST because imaging measures anatomy, whereas ctDNA reflects a short-half-life biological signal that can change within days to weeks [96]. Effective therapy typically reduces ctDNA quickly while radiographic regression may lag; conversely, rising ctDNA can indicate emerging resistance before measurable progression. These dynamics are most actionable in time-critical settings (e.g., PDAC and frail patients) and where resistance mechanisms have direct therapeutic consequences (e.g., anti-EGFR mCRC).

The appropriate clinical standard for ctDNA-guided switching is a randomized strategy trial comparing ctDNA-guided early switching versus standard imaging-guided management, with OS and/or QoL endpoints [48,100]. Observational associations, even when strong, are insufficient to justify routine switching because false positives could prematurely stop effective therapy and false negatives could delay necessary change. Ongoing platform trials in CRC offer a template for generating this level of evidence [101,102,103].

Until strategy-trial evidence matures across GI tumors, ctDNA is best used where it directly informs decisions: mRD risk-adapted adjuvant strategies in CRC (supported in stage II), resistance profiling that changes sequencing (including anti-EGFR rechallenge in mCRC), and trial enrichment to test intensification in mRD+ populations [104].

A pragmatic design principle is to prespecify thresholds for “molecular non-response” and require confirmatory testing to reduce false positives. For example, a predefined lack of ctDNA decline at 2–4 weeks could trigger repeat sampling and an early imaging checkpoint rather than immediate switching, balancing the potential value of earlier action with uncertainty [105].

At present, the most defensible clinical uses of early kinetics are trial enrichment (selecting high-risk biological non-responders), prompting earlier imaging when symptoms and biomarkers conflict, and enabling resistance-informed sequencing (most established in mCRC anti-EGFR). Routine ctDNA-guided switching across GI cancers remains premature without strategy-trial evidence [106,107].

Evidence linking early ctDNA kinetics to response and outcomes, and defining the methodological requirements for strategy trials, is supported by GI monitoring cohorts and meta-analyses [26,27,28].

## 11. Clinical Implementation and Pitfalls

Implementation is a clinical pathway, not an isolated test [40]. A minimum quality framework includes: (i) a predefined clinical question (mRD versus resistance monitoring), (ii) assay class selection (tumor-informed vs. tumor-agnostic; mutations vs. methylation/fragmentomics), (iii) standardized pre-analytics (e.g., collection tubes, processing time, and plasma volume), (iv) explicit CH/CHIP mitigation (paired WBC sequencing or validated filters), and (v) a pre-agreed action algorithm for both positive and negative results [41,108].

mRD introduces a new category of information: molecularly detectable, radiographically occult disease. Without preparation, ctDNA can become an anxiety amplifier [14]. Before testing, patients should understand what a positive result implies (high relapse risk and a defined plan), what a negative result does not imply (no guarantee of cure, especially in low-shedding tumors), and what the next steps will be for either outcome [14,109,110]. This “pre-test consent” mirrors best practice in genetic counseling because the psychological and decisional implications are real [41,49,109].

What to do with mRD+ and imaging-negative discordance. This scenario is increasingly common, especially with sensitive assays. A reflexive escalation to systemic therapy outside trials risks over-treatment; a reflexive “ignore” risks missed curative windows. A structured approach includes repeat ctDNA to confirm persistence/rise, review of CH/CHIP and sample quality, escalation of imaging modality where appropriate (e.g., liver MRI), and multidisciplinary review for oligometastatic salvage [49]. Trial enrollment for mRD-guided intervention should be prioritized when available [111,112].

When not to test. ctDNA/mRD should be avoided when interpretability is low or no action will follow, such as surveillance testing without a predefined intervention pathway, high-stakes adjuvant de-escalation based solely on a single negative result outside trials, or settings with poor plasma representation (peritoneal-only disease) when baseline ctDNA is non-measurable [47,49,50].

A realistic implementation pathway also requires institutional readiness. In practical terms, ctDNA should not be introduced as an isolated send-out test but as a governed clinical service involving oncology, molecular pathology, laboratory medicine, surgery, radiology, and multidisciplinary review. Minimum requirements include: a validated assay matched to the clinical indication, standardized pre-analytical handling, predefined sampling windows, explicit CH/CHIP mitigation, report formats that clearly state assay limitations, and prespecified downstream management pathways for both positive and negative results. At present, ctDNA testing has not been implemented as a routine standard-of-care pathway across our institutions for GI malignancies. In our view, broader adoption should remain selective and protocol-driven until these institutional prerequisites are in place and interventional evidence matures further outside the best-validated settings.

For *Cancers*/MDPI, reviewers will expect the explicit reporting of sampling windows relative to surgery, adjuvant therapy, or systemic treatment initiation, as well as assay class, CH/CHIP mitigation, and positivity definitions [108]. In narrative reviews, an explicit “interpretation framework” and tumor-specific implementation caveats add credibility and reduce the risk of over-claiming [14,49].

## 12. Research Priorities and Future Directions

The field is now at an inflection point: prognostic validity is increasingly established, while outcome-changing utility remains to be proven for most indications [45,64]. Six priorities should guide the next generation of GI ctDNA studies.

First, intensification trials in mRD+. In CRC, mRD+ defines a population with very high relapse risk; the question is whether escalation (novel agents, extended duration, or combination strategies) increases cure rates rather than merely postponing recurrence [112,113]. Similar logic applies to resected BTC and PDAC, where relapse is common and early [48,64].

Second, safe de-escalation trials in mRD-. Stage II CRC now has randomized long-term data supporting ctDNA-guided reduction in ACT exposure [48]. Stage III CRC and other GI tumors require caution because the cost of a false negative may be high [48,58]. A critical distinction is mRD- with documented baseline ctDNA detectability versus mRD- without baseline detectability [26,53]. Trial designs should stratify these groups rather than treating all negatives as equivalent [70,71,96].

Third, strategy trials for early switching based on ctDNA kinetics. Observational correlations between early ctDNA decline and outcomes are not sufficient [27,96]. Trials must randomize ctDNA-guided early switching versus standard imaging-guided management, with OS and patient-reported outcomes as endpoints [26]. This is most urgent in PDAC and advanced gastroesophageal cancers, where performance status can decline quickly and the window for later-line therapy is narrow [70,71,96].

Fourth, harmonized reporting standards. For mRD studies to be comparable, publications should report: assay class and signal type, plasma volume and cfDNA yield, marker count and aggregation strategy, LOD/LoQ and positivity criteria, CH/CHIP mitigation method, sampling timepoints, and how borderline results were adjudicated [114,115]. Without this, meta-analyses will continue to pool heterogeneous signals and clinicians will be left with “impressive hazard ratios” that do not translate into bedside rules [14,49].

Fifth, technology tailored to low-shedding tumors. In PDAC and HCC, methylation- and fragmentomics-based ctDNA approaches may improve sensitivity where mutation-only assays struggle [116,117]. Multimodal models integrating ctDNA with serum markers and imaging/radiomics are also of interest but remain investigational. Importantly, all such approaches require validation in the exact clinical window and decision context in which they are intended to be used (e.g., post-resection or post-ablative surveillance) and should not be assumed to be effective on the basis of early-detection performance alone.

Sixth, implementation science and equity. Turnaround time, cost, access to high-quality imaging, and availability of clinical trials will determine whether ctDNA improves outcomes or widens disparities [47]. Interventional designs should include pragmatic components (e.g., feasibility, adherence, and patient anxiety) because the burden of intensified surveillance or repeated blood draws is not negligible [41,117].

Ongoing ctDNA-guided strategy trials and implementation frameworks across GI cancers provide the roadmap for outcome-changing validation. A tumor-type-by-scenario clinical map of ctDNA use across gastrointestinal cancers (maturity, actionability, defensible interpretation, and key limitations) is summarized in Supplementary Table S6.

## 13. Limitations

This review has several limitations. First, it is a clinically oriented narrative synthesis rather than a formal systematic review or meta-analysis; we therefore did not perform an instrument-based risk-of-bias assessment for each included study. Second, the available literature is methodologically heterogeneous with respect to assay class, molecular substrate, sampling windows, positivity definitions, and clinical endpoints, which limits direct cross-study comparability. Third, the maturity of evidence is not uniform across GI malignancies: the strongest interventional and practice-relevant data are currently available in CRC, whereas in several other GI tumors, the evidence remains more observational and context-dependent. Finally, because this field is evolving rapidly, some implementation-oriented conclusions will require continued reappraisal as ongoing prospective strategy trials mature.

## 14. Conclusions

ctDNA is best understood not as a uniform marker but as a family of tumor- and context-specific molecular assays. The most mature and practice-informing evidence is currently available in CRC, where postoperative mRD strongly stratifies recurrence risk, randomized data support ctDNA-guided adjuvant strategies in stage II colon cancer, and resistance profiling can inform sequencing in metastatic disease. Outside CRC, positive ctDNA after curative-intent therapy is often clinically meaningful, whereas negative results remain strongly constrained by shedding biology, assay sensitivity, and disease context.

The next phase is interventional: converting prognostic signal into improved outcomes through mRD-guided intensification, safe de-escalation, and early-switching strategy trials. Until then, ctDNA should be implemented selectively and only alongside standard imaging, pathology, and multidisciplinary decision making, within governed pathways that include harmonized sampling/reporting standards and explicit CH/CHIP control.

## Figures and Tables

**Table 1:** ctDNA assay cheat-sheet: match the test to the clinical question.

Assay Class	Best Use	Actionability (How to Use Results)	If Negative, the Safest Wording Is…
TI multi-variant (tumor-informed)	Postop MRD, clearance on ACT, surveillance when trackable baseline is established	Rule-in: strong. Rule-out: conditional (depends on timing/QC/shedding). Trend: useful.	“No ctDNA detected above LOD at this timepoint with this assay. A rule-out conclusion requires appropriate timing, adequate sample/QC, and no low-shedding phenotype.”
TA mutation panel (tumor-agnostic SNV/indel)	Advanced CRC genotyping/resistance monitoring; MRD only with strict validation + CHIP control	Rule-in: moderate (must exclude CHIP). Rule-out: weak for MRD. Trend: supportive.	“No panel variants detected above LOD. In MRD, this does not exclude disease (may be below LOD or outside panel coverage).”
Methylation/fragmentomics (tissue-free signatures)	Selected very low TF settings only if validated for the same clinical window	Rule-in: moderate (platform-specific). Rule-out: variable and setting-dependent. Trend: supportive.	“No signal above the assay cut-off. Interpret in postop MRD only if validated for that setting; otherwise correlate with risk/imaging.”
ddPCR single hotspot	Rapid monitoring of a known dominant hotspot	Rule-in: strong for that hotspot. Rule-out: not applicable for MRD globally. Trend: strong for tracked clone.	“That hotspot is not detected above LOD; this is not equivalent to ‘no MRD’ and does not exclude other clones.”

Abbreviations: TI, tumor-informed; TA, tumor-agnostic; TF, tumor fraction; LOD, limit of detection; MRD, molecular residual disease.

**Table 2:** Tumor-specific ctDNA substrates, identification strategies, and principal interpretive implications across gastrointestinal cancers.

Tumor Type	Tumor-Specific ctDNA Substrate	Identification in Practice	Principal Clinical Value	Key Caveat
Colorectal cancer (CRC)	APC, TP53, KRAS/NRAS, BRAF, PIK3CA, SMAD4; patient-specific multi-variant signatures; emerging methylation-based signatures	Primarily tumor-informed tissue sequencing with personalized plasma tracking; alternatively, validated plasma-only mutation or methylation assays	Strongest evidence base among GI tumors. Postoperative ctDNA positivity reliably identifies patients at high risk of recurrence. Also useful for longitudinal MRD surveillance, assessment of adjuvant ctDNA clearance, and resistance monitoring in metastatic disease, including anti-EGFR rechallenge strategies	Best-performing GI setting, but a negative result still depends on sampling time, baseline detectability, analytical sensitivity, and exclusion of CHIP
Gastric/gastroesophageal adenocarcinoma	TP53-centered alterations with additional patient-specific genomic changes; personalized mutation panels or broader plasma NGS profiles	Usually tumor-informed assays or broad plasma NGS; serial sampling during perioperative therapy may improve interpretability	ctDNA positivity after perioperative therapy or surgery is clinically meaningful and associated with increased relapse risk. May support dynamic risk assessment and early relapse detection in selected patients	Plasma detectability is less consistent than in CRC; a negative result is not sufficiently reassuring to support treatment de-escalation
Esophageal cancer	Tumor-specific somatic variants, commonly including TP53 and other patient-level alterations; personalized panels in localized disease	Typically tumor-informed sequencing with plasma assessment after neoadjuvant chemoradiotherapy and/or surgery	Post-treatment ctDNA positivity suggests residual disease and higher recurrence risk. Potential utility in post-treatment surveillance is promising but remains investigational	ctDNA should be interpreted together with pathology, endoscopy, and imaging; it should not guide organ preservation decisions in isolation
Pancreatic ductal adenocarcinoma (PDAC)	KRAS hotspot mutations most commonly tracked; TP53, SMAD4, CDKN2A, and broader personalized variants in tumor-informed assays	Either KRAS-focused ddPCR when a hotspot is known or tumor-informed multi-variant assays when tissue is available	Postoperative ctDNA positivity is a strong indicator of micrometastatic disease. When baseline ctDNA is detectable, serial testing may help assess early treatment kinetics during systemic therapy	Low plasma shedding and inflammatory cfDNA background limit sensitivity; a negative result often remains non-informative
Biliary tract cancer (BTC)	IDH1, FGFR2 alterations/fusions, KRAS, TP53, and other tumor-specific genomic abnormalities	Often broad plasma NGS, particularly when tissue is limited; tumor-informed MRD strategies are emerging after resection	Clinically relevant both for prognostication and for detection of actionable or resistance alterations. Postoperative or persistent ctDNA positivity may identify patients at higher risk of recurrence	A dual prognostic and predictive role is attractive, but post-resection MRD data remain less mature than in CRC
Hepatocellular carcinoma (HCC)	TERT promoter, TP53, CTNNB1, and other tumor-associated alterations; increasingly, methylation- and fragmentomics-based signatures	Mutation-based assays and, in selected settings, methylation/fragmentomics approaches to improve signal detection in high-background-cfDNA conditions	Postoperative ctDNA positivity is associated with recurrence risk. ctDNA may also contribute to surveillance and relapse-risk stratification when incorporated into structured follow-up pathways	Cirrhosis, necroinflammation, and high background cfDNA reduce rule-out value; a negative result should not justify surveillance de-escalation

Footnote: This table is intended as a tumor-specific interpretive synthesis of the cited literature rather than as a study-level evidence summary. Abbreviations: BTC, biliary tract cancer; cfDNA, cell-free DNA; CHIP, clonal hematopoiesis of indeterminate potential; CRC, colorectal cancer; ctDNA, circulating tumor DNA; ddPCR, droplet digital polymerase chain reaction; EGFR, epidermal growth factor receptor; GI, gastrointestinal; HCC, hepatocellular carcinoma; MRD, molecular residual disease; NGS, next-generation sequencing; PDAC, pancreatic ductal adenocarcinoma.

**Table 3:** ctDNA-defined mRD trajectories after curative-intent resection of CRC: practical interpretation pathways and guardrails.

mRD (ctDNA) Trajectory	Interpretation and Risk	Recommended Pathway (Minimum Standard)	Guardrails (Pitfalls + Must-Checks)
A. Persistently mRD− (serially negative)	No ctDNA detected in this assay at these timepoints. Can still be low-shedding/compartmental disease. Risk: low–intermediate (modified by stage/pathology).	Standard surveillance as per guidelines. Adjuvant decisions by stage/risk standards (do not omit standard ACT in higher-risk settings outside trials). If high-risk biology or low-shedding phenotype suspected, prioritize imaging strategy and clinical judgement.	Avoid: “cure” statements; de-escalation based on one negative test. Must-check: baseline detectability/trackable targets; sampling window; plasma volume and pre-analytics; assay class (TI/TA) + LOD; low-shedding phenotypes (mucinous/peritoneal-only/low burden).
B. Postop mRD+ → sustained clearance during/after ACT	Treatment-sensitive micrometastatic disease with molecular response. Risk: intermediate (best among initially positive).	Complete planned ACT (if indicated). Confirm clearance with EOT ctDNA + one confirmatory sample (typically 4–8 weeks later). Then return to standard surveillance; manage discordant symptoms/imaging clinically.	Avoid: escalation “just in case” with sustained clearance and imaging−; overcalling borderline negatives. Must-check: consistent assay over time; explicit definition of clearance (e.g., 2 consecutive negatives); comparable pre-analytics between timepoints.
C. Persistently mRD+ despite ACT/mRD+ at EOT	Ongoing systemic residual disease; often resistant biology. Risk: very high.	If low-level/borderline: repeat ctDNA in 2–4 weeks to confirm persistence/trend. In parallel: review for artefact/CHIP (esp. TA panels), and then trigger directed imaging + MDT. If imaging−, follow predefined “mRD+/imaging−” plan (scheduled repeat ctDNA + timed imaging). Prefer trial/protocolized intensification when available.	Avoid: ad hoc therapy without MDT and a plan, especially when imaging−. Must-check: CHIP mitigation (TA panels); sample quality/QC; confounders (recent surgery/inflammation); consider compartmental disease when plasma signal is discordant.
D. Conversion mRD− → mRD+ during surveillance	Emerging molecular relapse, often preceding radiology. Risk: high.	Confirm promptly (repeat ctDNA ~2–4 weeks) unless analytically unequivocal. Escalate site-directed imaging and MDT review (salvageable oligometastatic vs. systemic pattern). If imaging−, use the predefined “mRD+/imaging−” pathway—avoid random testing.	Avoid: “unstructured escalation” from a single new positive; treatment switching without confirmation (unless clinically urgent + strong analytical certainty). Must-check: predefined action plan for (+)/(−) before ordering; consistent intervals/platform; artefact/CHIP review, especially with TA mutation panels.

Abbreviations: ACT, adjuvant chemotherapy; EOT, end of treatment; MDT, multidisciplinary team; LOD, limit of detection; TI, tumor-informed; TA, tumor-agnostic.

**Table 4:** ctDNA in anti-EGFR-treated mCRC: decision triggers, actionability, and guardrails.

Scenario	What to Track	Decision Trigger (Minimal Action)	Actionability	Guardrails (Must-Checks)
On anti-EGFR therapy: suspected loss of benefit	Rising/appearing resistance categories (RAS-pathway, BRAF, EGFR-ECD; platform-dependent)	New or rising resistance signal on a sample with demonstrable ctDNA → bring imaging forward and discuss next-line strategy in MDT (do not wait for overt RECIST progression if clinically deteriorating). Borderline/low TF → repeat ctDNA in 2–4 weeks before acting.	Rule-in resistance: supportive to strong (for timing of imaging/strategy planning). Trend: useful if consistent sampling and assay.	No automatic switching based on ctDNA alone. “Negative resistance” ≠ no resistance (low TF, limited panel). Document: baseline ctDNA detectability/TF, LOD, panel breadth, and CHIP mitigation where relevant.
Anti-EGFR rechallenge after a drug holiday	Absence/decay of prior resistance clones (same resistance categories as above)	No detectable resistance above LOD and ctDNA is measurable/trackable → reasonable to proceed with rechallenge (with early clinical/radiologic reassessment plan). Borderline low-level positivity → repeat ctDNA and decide by predefined threshold.	Patient selection: strongest use case (biomarker-enriched rechallenge).	Not a guarantee; does not replace RECIST/clinical assessment. If ctDNA is undetectable overall, interpret “no resistance” cautiously. Predefine what you will do for: negative/borderline/positive. Keep the same platform and thresholds.

Abbreviations: anti-EGFR, cetuximab/panitumumab; TF, tumor fraction; LOD, limit of detection; RECIST, radiologic response criteria; MDT, multidisciplinary team. Framework intended for clinical interpretation of the cited evidence rather than as a formal guideline algorithm.

## Data Availability

No new data were created or analyzed in this study.

## Supplementary Materials

The following supporting information can be downloaded at: https://www.mdpi.com/article/10.3390/cancers18061014/s1, Supplementary Table S1: Full reproducible PubMed/MEDLINE search strategy; Supplementary Table S2: Representative quantitative anchors for ctDNA-based mRD assessment and monitoring across major gastrointestinal cancers; Supplementary Table S3: Additional studies and reporting framework (checklist for comparability); Supplementary Table S4: Clonal hematopoiesis (CH/CHIP) in ctDNA testing: recognition and mitigation of false positives; Supplementary Table S5: Pre-analytics and QC for ctDNA (MRD and monitoring): minimum standards, rejection rules, and reporting; Supplementary Table S6: Biological limitations of ctDNA: phenotype matrix and mitigation strategies; Supplementary Table S7: ctDNA monitoring: suggested schedules, trend interpretation, and clinical responses; Supplementary Table S8: Clinical map of ctDNA use in gastrointestinal cancers: maturity, actionability, and limitations; Supplementary Table S9: Landmark published and ongoing ctDNA studies across major gastrointestinal cancers. References [118,119,120,121] are cited in Supplementary Materials.

## Abbreviations

The following abbreviations are used in this manuscript:

ACT	adjuvant chemotherapy
AFP	alpha-fetoprotein
BRAF	B-Raf proto-oncogene (serine/threonine kinase)
BTC	biliary tract cancer
CA19-9	carbohydrate antigen 19-9
CEA	carcinoembryonic antigen
cfDNA	cell-free DNA
CH	clonal hematopoiesis
CHIP	clonal hematopoiesis of indeterminate potential
CRC	colorectal cancer
ctDNA	circulating tumor DNA
ddPCR	droplet digital PCR
DFS	disease-free survival
EGFR	epidermal growth factor receptor
GEJ	gastroesophageal junction
GI	gastrointestinal
HCC	hepatocellular carcinoma
IDH1	isocitrate dehydrogenase 1
LOD	limit of detection
LoQ	limit of quantification
mCRC	metastatic colorectal cancer
MRD	minimal/molecular residual disease
mRD	molecular residual disease
MSI	microsatellite instability
nCRT	neoadjuvant chemoradiotherapy
OS	overall survival
PDAC	pancreatic ductal adenocarcinoma
PET	positron emission tomography
PET/CT	positron emission tomography/computed tomography
PFS	progression-free survival
RAS	RAS oncogene family (KRAS/NRAS/HRAS)
RECIST	Response Evaluation Criteria in Solid Tumors

## References

[1] Nors J., Gotschalck K.A., Erichsen R., Andersen C. (2024). Incidence of Recurrence and Time to Recurrence in Stage I to III Colorectal Cancer: A Nationwide Danish Cohort Study. JAMA Oncol..

[2] Qaderi S.M., Galjart B., Verhoef C., Slooter G.D., Koopman M., Verhoeven R.H.A., de Wilt J.H.W., van Erning F.N. (2021). Disease recurrence after colorectal cancer surgery in the modern era: A population-based study. Int. J. Color. Dis..

[3] Locker G.Y., Hamilton S., Harris J., Jessup J.M., Kemeny N., Macdonald J.S., Somerfield M.R., Hayes D.F., Bast R.C. (2006). ASCO 2006 update of recommendations for the use of tumor markers in gastrointestinal cancer. J. Clin. Oncol..

[4] Lee T., Teng T.Z.J., Shelat V.G. (2020). Carbohydrate antigen 19-9—Tumor marker: Past, present, and future. World J. Gastrointest. Surg..

[5] Eisenhauer E.A., Therasse P., Bogaerts J., Schwartz L.H., Sargent D., Ford R., Dancey J., Arbuck S., Gwyther S., Mooney M. (2009). New response evaluation criteria in solid tumours: Revised RECIST guideline (version 1.1). Eur. J. Cancer.

[6] Frank M.S., Andersen C.S.A., Ahlborn L.B., Pallisgaard N., Bodtger U., Gehl J. (2022). Circulating tumor DNA monitoring reveals molecular progression before radiologic progression in a real-life cohort of patients with advanced non–small cell lung cancer. Cancer Res. Commun..

[7] Marano L., Verre L., Carbone L., Poto G.E., Fusario D., Venezia D.F., Calomino N., Kaźmierczak-Siedlecka K., Polom K., Marrelli D. (2023). Current Trends in Volume and Surgical Outcomes in Gastric Cancer. J. Clin. Med..

[8] Sánchez-Herrero E., Serna-Blasco R., Robado de Lope L., González-Rumayor V., Romero A., Provencio M. (2022). Circulating Tumor DNA as a Cancer Biomarker. Front. Oncol..

[9] Siravegna G., Marsoni S., Siena S., Bardelli A. (2017). Integrating liquid biopsies into the management of cancer. Nat. Rev. Clin. Oncol..

[10] Topham J.T., O’Callaghan C.J., Feilotter H., Kennecke H.F., Lee Y.S., Li W., Banks K.C., Quinn K., Renouf D.J., Jonker D.J. (2023). Circulating Tumor DNA Identifies Diverse Landscape of Acquired Resistance to Anti–Epidermal Growth Factor Receptor Therapy in Metastatic Colorectal Cancer. J. Clin. Oncol..

[11] Tie J., Cohen J.D., Lahouel K., Lo S.N., Wang Y., Kosmider S., Wong R., Shapiro J., Lee M., Harris S. (2022). Circulating Tumor DNA Analysis Guiding Adjuvant Therapy in Stage II Colon Cancer. N. Engl. J. Med..

[12] Soveri L.M., Lamminmäki A., Hänninen U.A., Karhunen M., Bono P., Osterlund P. (2019). Long-term neuropathy and quality of life in colorectal cancer patients treated with oxaliplatin containing adjuvant chemotherapy. Acta Oncol..

[13] Teng C., Cohen J., Egger S., Blinman P.L., Vardy J.L. (2022). Systematic review of long-term chemotherapy-induced peripheral neuropathy following adjuvant oxaliplatin for colorectal cancer. Support. Care Cancer.

[14] Merker J.D., Oxnard G.R., Compton C., Diehn M., Hurley P., Lazar A.J., Lindeman N., Lockwood C.M., Rai A.J., Schilsky R.L. (2018). Circulating Tumor DNA Analysis in Patients with Cancer: ASCO and CAP Joint Review. J. Clin. Oncol..

[15] Dasari A., Morris V.K., Allegra C.J., Atreya C., Benson A.B., Boland P., Chung K., Copur M.S., Corcoran R.B., Deming D.A. (2020). ctDNA applications and integration in colorectal cancer: An NCI Colon and Rectal–Anal Task Forces whitepaper. Nat. Rev. Clin. Oncol..

[16] Kasi P.M., Fehringer G., Taniguchi H., Starling N., Nakamura Y., Kotani D., Powles T., Li B.T., Pusztai L., Aushev V.N. (2022). Impact of circulating tumor DNA-based detection of minimal residual disease: Trial design and clinical pathways. JCO Precis. Oncol..

[17] Heitzer E., Haque I.S., Roberts C.E.S., Speicher M.R. (2019). Current and future perspectives of liquid biopsies in genomics-driven oncology. Nat. Rev. Genet..

[18] Newman A.M., Lovejoy A.F., Klass D.M., Kurtz D.M., Chabon J.J., Scherer F., Stehr H., Liu C.L., Bratman S.V., Say C. (2016). Integrated digital error suppression for improved detection of circulating tumor DNA. Nat. Biotechnol..

[19] Razavi P., Li B.T., Brown D.N., Jung B., Hubbell E., Shen R., Abida W., Juluru K., De Bruijn I., Hou C. (2019). High-intensity sequencing reveals the sources of plasma circulating cell-free DNA variants. Nat. Med..

[20] Bettegowda C., Sausen M., Leary R.J., Kinde I., Wang Y., Agrawal N., Bartlett B.R., Wang H., Luber B., Alani R.M. (2014). Detection of circulating tumor DNA in early- and late-stage human malignancies. Sci. Transl. Med..

[21] Cristiano S., Leal A., Phallen J., Fiksel J., Adleff V., Bruhm D.C., Jensen S.Ø., Medina J.E., Hruban C., White J.R. (2019). Genome-wide cell-free DNA fragmentation patterns and cancer detection. Nature.

[22] Sahin I.H., Lin Y., Yothers G., Lucas P.C., Deming D., George T.J., Kopetz S., Lieu C.H., Dasari A. (2022). Minimal Residual Disease-Directed Adjuvant Therapy for Patients with Early-Stage Colon Cancer: CIRCULATE-US. Oncology.

[23] Nakamura Y., Watanabe J., Akazawa N., Hirata K., Kataoka K., Yokota M., Kato K., Kotaka M., Kagawa Y., Yeh K.H. (2024). ctDNA-based molecular residual disease and survival in resectable colorectal cancer. Nat. Med..

[24] Tie J., Wang Y., Tomasetti C., Li L., Springer S., Kinde I., Silliman N., Tacey M., Wong H.L., Christie M. (2016). Circulating tumor DNA analysis detects minimal residual disease and predicts recurrence in stage II colon cancer. Sci. Transl. Med..

[25] Reinert T., Henriksen T.V., Christensen E., Sharma S., Salari R., Sethi H., Knudsen M., Nordentoft I., Wu H.T., Tin A.S. (2019). Analysis of plasma cell-free DNA by ultradeep sequencing in patients with stages I–III colorectal cancer. JAMA Oncol..

[26] Tatalovic S., Doleschal B., Kupferthaler A., Grundner S., Burghofer J., Webersinke G., Schwendinger S., Jukic E., Zschocke J., Danhel L. (2024). Circulating Tumor DNA (ctDNA) Dynamics Predict Early Response to Treatment in Metastasized Gastroesophageal Cancer After 2 Weeks of Systemic Treatment. Cancers.

[27] Parikh A.R., Mojtahed A., Schneider J.L., Kanter K., Van Seventer E.E., Fetter I.J., Thabet A., Fish M.G., Teshome B., Fosbenner K. (2020). Serial ctDNA Monitoring to Predict Response to Systemic Therapy in Metastatic Gastrointestinal Cancers. Clin. Cancer Res..

[28] Holz A., Paul B., Zapf A., Pantel K., Joosse S.A. (2025). Circulating tumor DNA as prognostic marker in patients with metastatic colorectal cancer: A systematic review and meta-analysis. Cancer Treat. Rev..

[29] ClinicalTrials.gov Colon Adjuvant Chemotherapy Based on Evaluation of Residual Disease (CIRCULATE-US; NRG-GI008). NCT05174169. NCT05174169.

[30] Taniguchi H., Nakamura Y., Kotani D., Yukami H., Mishima S., Sawada K., Shirasu H., Ebi H., Yamanaka T., Aleshin A. (2021). CIRCULATE-Japan: Circulating tumor DNA-guided adaptive platform trials to refine adjuvant therapy for colorectal cancer. Cancer Sci..

[31] ClinicalTrials.gov CIRCULATE Trial (ctDNA-Guided Strategies After Colon Tumor Surgery). NCT04120701. NCT04120701.

[32] Lordick F., Carneiro F., Cascinu S., Fleitas T., Haustermans K., Piessen G., Vogel A., Smyth E.C., ESMO Guidelines Committee (2022). Gastric cancer: ESMO Clinical Practice Guideline for diagnosis, treatment and follow-up. Ann Oncol..

[33] Conroy T., Desseigne F., Ychou M., Bouché O., Guimbaud R., Bécouarn Y., Adenis A., Raoul J.L., Gourgou-Bourgade S., de la Fouchardière C. (2011). FOLFIRINOX versus gemcitabine for metastatic pancreatic cancer. N. Engl. J. Med..

[34] Finn R.S., Qin S., Ikeda M., Galle P.R., Ducreux M., Kim T.Y., Kudo M., Breder V., Merle P., Kaseb A.O. (2020). Atezolizumab plus bevacizumab in unresectable hepatocellular carcinoma. N. Engl. J. Med..

[35] Corcoran R.B., Chabner B.A. (2018). Application of Cell-free DNA Analysis to Cancer Treatment. N. Engl. J. Med..

[36] Schöpfer L., Zisser L., Oberndorfer F., Schiefer A.I., Compérat E.M., Müllauer L., Oszwald A. (2025). Validation and clinical evaluation of a comprehensive circulating tumor DNA assay for genomic profiling in solid tumors. BMC Cancer.

[37] Andersen L., Kisistók J., Henriksen T.V., Bramsen J.B., Reinert T., Øgaard N., Mattesen T.B., Birkbak N.J., Andersen C.L. (2024). Exploring the biology of ctDNA release in colorectal cancer. Eur. J. Cancer.

[38] Mahuron K.M., Fong Y. (2024). Applications of Liquid Biopsy for Surgical Patients with Cancer: A Review. JAMA Surg..

[39] Abdo T., Alhalabi A., Yaghi S., Aloran M., Li Y., Herrán M., Tfayli R., Samuel T.A., Nahleh Z. (2026). Minimal residual disease in solid tumors: Clinical applications and future directions. Cancer.

[40] Driussi A., Lamaze F.C., Kordahi M., Armero V.S., Gaudreault N., Orain M., Enlow W., Abbosh C., Hodgson D., Dasgupta A. (2025). Clinicopathological Predictors of the Presence of Blood Circulating Tumor DNA in Early-Stage Non-Small Cell Lung Cancers. Mod. Pathol..

[41] Krebs M.G., Malapelle U., André F., Paz-Ares L., Schuler M., Thomas D.M., Vainer G., Yoshino T., Rolfo C. (2022). Practical Considerations for the Use of Circulating Tumor DNA in the Treatment of Patients with Cancer: A Narrative Review. JAMA Oncol..

[42] Chen H., Zhou Q. (2023). Detecting liquid remnants of solid tumors treated with curative intent: Circulating tumor DNA as a biomarker of minimal residual disease (Review). Oncol. Rep..

[43] Martelli V., Vidal J., Salvans S., Fernández C., Badia-Ramentol J., Linares J., Jiménez M., Sibilio A., Gibert J., Pérez M. (2025). Liquid Biopsy in Peritoneal Carcinomatosis from Colorectal Cancer: Current Evidence and Future Perspectives. Cancers.

[44] van der Sluis K., van Sandick J.W., Vollebergh M.A., van Dieren J.M., Hugen N., Hartemink K.J., Veenhof A.A.F.A., Verhoeven E., van den Berg J.G., Snaebjornsson P. (2024). Improving diagnostic accuracy of identifying gastric cancer patients with peritoneal metastases: Tumor-guided cell-free DNA analysis of peritoneal fluid. Oncogene.

[45] Hoang T., Choi M.K., Oh J.H., Kim J. (2025). Utility of circulating tumor DNA to detect minimal residual disease in colorectal cancer: A systematic review and network meta-analysis. Int. J. Cancer.

[46] Buonopane I.R., Saldanha E.F., de Menezes J.S.A., da Conceição L.D., Reis C.M.P., Leite L.F., Francischetto T., Peixoto R.D., Biachi de Castria T. (2026). Circulating tumour DNA for a minimal residual disease assessment and recurrence risk in hepatocellular carcinoma: A systematic review and meta-analysis. Br. J. Cancer.

[47] Carr D.J., Welch H.G. (2023). Assessing the Clinical Utility of Liquid Biopsies Across 5 Potential Indications From Therapy Selection to Population Screening: A Review. JAMA Intern. Med..

[48] Tie J., Wang Y., Loree J.M., Cohen J.D., Wong R., Price T., Tebbutt N.C., Gebski V., Espinoza D., Burge M. (2025). Circulating tumor DNA-guided adjuvant therapy in locally advanced colon cancer: The randomized phase 2/3 DYNAMIC-III trial. Nat. Med..

[49] Pascual J., Attard G., Bidard F.C., Curigliano G., De Mattos-Arruda L., Diehn M., Italiano A., Lindberg J., Merker J.D., Montagut C. (2022). ESMO recommendations on the use of circulating tumour DNA assays for patients with cancer: A report from the ESMO Precision Medicine Working Group. Ann. Oncol..

[50] Cohen S.A., Liu M.C., Aleshin A. (2023). Practical recommendations for using ctDNA in clinical decision making. Nature.

[51] Xia L., Pu Q., Kang R., Mei J., Li L., Yang Y., Deng S., Feng G., Deng Y., Gan F. (2025). Dynamic ctDNA informs whole-course postoperative precise management of NSCLC (LUNGCA study). J. Natl. Cancer Inst..

[52] Tie J., Wang Y., Lo S.N., Lahouel K., Cohen J.D., Wong R., Shapiro J.D., Harris S.J., Khattak A., Burge M.E. (2025). Circulating tumor DNA analysis guiding adjuvant therapy in stage II colon cancer: 5-year outcomes of the randomized DYNAMIC trial. Nat. Med..

[53] Parikh A.R., Van Seventer E.E., Siravegna G., Hartwig A.V., Jaimovich A., He Y., Kanter K., Fish M.G., Fosbenner K.D., Miao B. (2021). Minimal Residual Disease Detection using a Plasma-only Circulating Tumor DNA Assay in Patients with Colorectal Cancer. Clin. Cancer Res..

[54] Cohen S.A., Aushev V.N., Laliotis G., Jabbal I.S., Nagarajan A., Wang C., Fakih M., Sharif S., Alyunis F., Tejani M.A. (2025). Real-World Monitoring of ctDNA Reliably Predicts Cancer Recurrence and Treatment Efficacy in Patients with Resected Stages I-III Colon Cancer. Ann. Surg..

[55] Faulkner L.G., Howells L.M., Pepper C., Shaw J.A., Thomas A.L. (2023). The utility of ctDNA in detecting minimal residual disease following curative surgery in colorectal cancer: A systematic review and meta-analysis. Br. J. Cancer.

[56] Nakamura Y., Tsukada Y., Matsuhashi N., Murano T., Shiozawa M., Takahashi Y., Oki E., Goto M., Kagawa Y., Kanazawa A. (2024). Colorectal Cancer Recurrence Prediction Using a Tissue-Free Epigenomic Minimal Residual Disease Assay. Clin. Cancer Res..

[57] Sinicrope F.A., Segovia D., Sharma N., Alberts S.R., Hardin A., Rich T., Shi Q. (2026). Tissue-Free Circulating Tumor DNA Assay and Patient Outcome in a Phase III Trial of FOLFOX-Based Adjuvant Chemotherapy (Alliance N0147). J. Clin. Oncol..

[58] Caughey B.A., Parikh A.R. (2024). ctDNA/MRD Testing for Colon Cancer: A Work in Progress or Ready for Prime-Time Standard of Care?. J. Natl. Compr. Cancer Netw..

[59] Slater S., Bryant A., Aresu M., Begum R., Chen H.C., Peckitt C., Lazaro-Alcausi R., Carter P., Anandappa G., Khakoo S. (2024). Tissue-Free Liquid Biopsies Combining Genomic and Methylation Signals for Minimal Residual Disease Detection in Patients with Early Colorectal Cancer from the UK TRACC Part B Study. Clin. Cancer Res..

[60] Loft M., To Y.H., Gibbs P., Tie J. (2023). Clinical application of circulating tumour DNA in colorectal cancer. Lancet Gastroenterol. Hepatol..

[61] Zaanan A., Bergen E.S., Evesque L., Meurisse A., Artru P., Lecomte T., Bouché O., Lepère C., Ambrosini M., Coriat R. (2025). Circulating tumor DNA driving anti-EGFR rechallenge therapy in metastatic colorectal cancer: The RASINTRO prospective multicenter study. J. Natl. Cancer Inst..

[62] Ando K., Hamabe A., Nakamura Y., Watanabe J., Hirata K., Kataoka K., Miyo M., Kato K., Akazawa N., Kagawa Y. (2026). Molecular Residual Disease and Recurrence in Rectal Cancer Patients Undergoing Upfront Surgery: A Prospective Cohort Study. Ann. Surg..

[63] Henriksen T.V., Demuth C., Frydendahl A., Nors J., Nesic M., Rasmussen M.H., Reinert T., Larsen O.H., Jaensch C., Løve U.S. (2024). Unraveling the potential clinical utility of circulating tumor DNA detection in colorectal cancer-evaluation in a nationwide Danish cohort. Ann. Oncol..

[64] Lee M.S., Kaseb A.O., Pant S. (2023). The Emerging Role of Circulating Tumor DNA in Non-Colorectal Gastrointestinal Cancers. Clin. Cancer Res..

[65] Huffman B.M., Aushev V.N., Budde G.L., Chao J., Dayyani F., Hanna D., Botta G.P., Catenacci D.V.T., Maron S.B., Krinshpun S. (2022). Analysis of Circulating Tumor DNA to Predict Risk of Recurrence in Patients with Esophageal and Gastric Cancers. JCO Precis. Oncol..

[66] Openshaw M.R., Suwaidan A.A., Ottolini B., Fernandez-Garcia D., Richards C.J., Page K., Guttery D.S., Thomas A.L., Shaw J.A. (2020). Longitudinal monitoring of circulating tumour DNA improves prognostication and relapse detection in gastroesophageal adenocarcinoma. Br. J. Cancer.

[67] Azad T.D., Chaudhuri A.A., Fang P., Qiao Y., Esfahani M.S., Chabon J.J., Hamilton E.G., Yang Y.D., Lovejoy A., Newman A.M. (2020). Circulating Tumor DNA Analysis for Detection of Minimal Residual Disease After Chemoradiotherapy for Localized Esophageal Cancer. Gastroenterology.

[68] Chen B., Liu S., Zhu Y., Wang R., Cheng X., Chen B., Dragomir M.P., Zhang Y., Hu Y., Liu M. (2024). Predictive role of ctDNA in esophageal squamous cell carcinoma receiving definitive chemoradiotherapy combined with toripalimab. Nat. Commun..

[69] Eyck B.M., Jansen M.P., Noordman B.J., Atmodimedjo P.N., van der Wilk B.J., Martens J.W., Helmijr J.A., Beaufort C.M., Mostert B., Doukas M. (2023). Detection of circulating tumour DNA after neoadjuvant chemoradiotherapy in patients with locally advanced oesophageal cancer. J. Pathol..

[70] Sahwan O., Batha L., Jamal F., Sonbol M.B. (2025). Circulating Tumor DNA (ctDNA) in Gastroesophageal Adenocarcinoma (GEA): Evidence and Emerging Applications. Cancers.

[71] Zaanan A., Didelot A., Broudin C., Laliotis G., Spickard E., Dutta P., Saltel-Fulero A., Sullo F.G., Pizzamiglio M., Mariani A. (2025). Longitudinal circulating tumor DNA analysis during treatment of locally advanced resectable gastric or gastroesophageal junction adenocarcinoma: The PLAGAST prospective biomarker study. Nat. Commun..

[72] Hu Q., Kimura Y., Ikeda S., Tanaka Y., Nakanoko T., Ota M., Yoshizumi T., Eto M., Oki E. (2025). Circulating tumor DNA monitoring detects minimal residual disease and predicts outcomes in patients with esophageal adenocarcinoma or squamous cell carcinoma after esophagectomy. BJC Rep..

[73] Groot V.P., Mosier S., Javed A.A., Teinor J.A., Gemenetzis G., Ding D., Haley L.M., Yu J., Burkhart R.A., Hasanain A. (2019). Circulating Tumor DNA as a Clinical Test in Resected Pancreatic Cancer. Clin. Cancer Res..

[74] Borges F.C., Pinto M.S., Borges M.F., João A.A., Francisco E., Sousa M., Aral M., Oliveira V., Cunha J.F., Mehrabi A. (2026). The Role of Circulating Tumor DNA in Surgical Management of Pancreatic Cancer: Systematic Review and Meta-Analysis. Ann. Surg..

[75] Guo M.Z., Sachidanand A.S., Nguyen T., Patel S.D., Burns W.R., Burkhart R., Le D.T., Lafaro K., Bever K.M., Pishvaian M.J. (2026). Postsurgical circulating tumor DNA as a prognostic biomarker for relapse of resected pancreatic ductal adenocarcinoma. J. Gastrointest. Surg..

[76] Aaquist T., Henriksen T.V., Mariager Jakobsen L.L., Fristrup C.W., Pfeiffer P., de Stricker K., Sparrelid E., Moro C.F., Mortensen F., Knudsen A.R. (2026). Circulating tumour DNA as a prognostic tool for surgically treated pancreatic ductal adenocarcinoma. Hum. Pathol..

[77] Lee B., Lipton L., Cohen J., Tie J., Javed A.A., Li L., Goldstein D., Burge M., Cooray P., Nagrial A. (2019). Circulating tumor DNA as a potential marker of adjuvant chemotherapy benefit following surgery for localized pancreatic cancer. Ann. Oncol..

[78] Kitahata Y., Kawai M., Hirono S., Okada K.I., Miyazawa M., Motobayashi H., Ueno M., Hayami S., Miyamoto A., Yamaue H. (2022). Circulating Tumor DNA as a Potential Prognostic Marker in Patients with Borderline-Resectable Pancreatic Cancer Undergoing Neoadjuvant Chemotherapy Followed by Pancreatectomy. Ann. Surg. Oncol..

[79] Steiniche M.M., Lindgaard S.C., Chen I.M., Johansen J.S., Andersen R.F., Hansen T.F., Jensen L.H., Rasmussen L.S., Johansen M.W., Ladekarl M. (2025). The Prognostic Impact of Early ctDNA Kinetics in Metastatic Pancreatic Cancer Using the ctDNA-RECIST. Clin. Cancer Res..

[80] Pietrasz D., Wang-Renault S., Taieb J., Dahan L., Postel M., Durand-Labrunie J., Le Malicot K., Mulot C., Rinaldi Y., Phelip J.M. (2022). Prognostic value of circulating tumour DNA in metastatic pancreatic cancer patients: Post-hoc analyses of two clinical trials. Br. J. Cancer.

[81] Hata T., Mizuma M., Motoi F., Ohtsuka H., Nakagawa K., Morikawa T., Unno M. (2023). Prognostic impact of postoperative circulating tumor DNA as a molecular minimal residual disease marker in patients with pancreatic cancer undergoing surgical resection. J. Hepatobiliary Pancreat. Sci..

[82] Ueberroth B.E., Jones J.C., Bekaii-Saab T.S. (2022). Circulating tumor DNA (ctDNA) to evaluate minimal residual disease (MRD), treatment response, and posttreatment prognosis in pancreatic adenocarcinoma. Pancreatology.

[83] Watanabe F., Suzuki K., Tamaki S., Abe I., Endo Y., Takayama Y., Ishikawa H., Kakizawa N., Saito M., Futsuhara K. (2019). Longitudinal monitoring of KRAS-mutated circulating tumor DNA enables the prediction of prognosis and therapeutic responses in patients with pancreatic cancer. PLoS ONE.

[84] Lapin M., Edland K.H., Tjensvoll K., Oltedal S., Austdal M., Garresori H., Rozenholc Y., Gilje B., Nordgård O. (2023). Comprehensive ctDNA Measurements Improve Prediction of Clinical Outcomes and Enable Dynamic Tracking of Disease Progression in Advanced Pancreatic Cancer. Clin. Cancer Res..

[85] Botta G.P., Abdelrahim M., Drengler R.L., Aushev V.N., Esmail A., Laliotis G., Brewer C.M., George G.V., Abbate S.M., Chandana S.R. (2024). Association of personalized and tumor-informed ctDNA with patient survival outcomes in pancreatic adenocarcinoma. Oncologist.

[86] Park Y., Kim K.S., Jo H., Kim H.P., Kyung D.S., Yoo Y., Min S.K., Nam E.M., Kim K. (2026). Feasibility of tumor-informed circulating tumor DNA for detecting minimal residual disease in surgically resected biliary tract cancer. PLoS ONE.

[87] Yu J., He A.R., Ouf M., Mehta R., Anaya D.A., Denbo J., Bridges C., Tin A., Aushev V.N., Palsuledesai C.C. (2025). Detecting Early Recurrence with Circulating Tumor DNA in Stage I-III Biliary Tract Cancer After Curative Resection. JCO Precis. Oncol..

[88] Gögenur M., Hadi N.A., Qvortrup C., Andersen C.L., Gögenur I. (2022). ctDNA for Risk of Recurrence Assessment in Patients Treated with Neoadjuvant Treatment: A Systematic Review and Meta-analysis. Ann. Surg. Oncol..

[89] Berchuck J.E., Facchinetti F., DiToro D.F., Baiev I., Majeed U., Reyes S., Chen C., Zhang K., Sharman R., Uson Junior P.L.S. (2022). The clinical landscape of cell-free DNA alterations in 1671 patients with advanced biliary tract cancer. Ann. Oncol..

[90] de Scordilli M., Bortolot M., Torresan S., Noto C., Rota S., Di Nardo P., Fumagalli A., Guardascione M., Ongaro E., Foltran L. (2025). Precision oncology in biliary tract cancer: The emerging role of liquid biopsy. ESMO Open..

[91] Scott A.J., Sharman R., Shroff R.T. (2022). Precision Medicine in Biliary Tract Cancer. J. Clin. Oncol..

[92] Lamarca A., Kapacee Z., Breeze M., Bell C., Belcher D., Staiger H., Taylor C., McNamara M.G., Hubner R.A., Valle J.W. (2020). Molecular Profiling in Daily Clinical Practice: Practicalities in Advanced Cholangiocarcinoma and Other Biliary Tract Cancers. J. Clin. Med..

[93] Yoo C., Jeong H., Jeong J.H., Kim K.P., Lee S., Ryoo B.Y., Hwang D.W., Lee J.H., Moon D.B., Kim K.H. (2025). Circulating tumor DNA status and dynamics predict recurrence in patients with resected extrahepatic cholangiocarcinoma. J. Hepatol..

[94] Abou-Alfa G.K., Sahai V., Hollebecque A., Vaccaro G., Melisi D., Al-Rajabi R., Paulson A.S., Borad M.J., Gallinson D., Murphy A.G. (2020). Pemigatinib for previously treated, locally advanced or metastatic cholangiocarcinoma: A multicentre, open-label, phase 2 study. Lancet Oncol..

[95] Rimassa L., Lamarca A., O’Kane G.M., Edelie J., McNamara M.G., Vofel A., Fassan M., Former A., Kendall T., Adeca J. (2025). New systemic treatment paradigms in advanced biliary tract cancer and variations in patient access across Europe. Lancet Reg. Health Eur..

[96] Mehta R., Rivero-Hinojosa S., Dayyani F., Ferguson J., Shariff B., Aushev V.N., Budde G.L., Ortiz J.B., George G.V., Sharma S. (2026). Circulating tumor DNA informs clinical practice in patients with recurrent/metastatic gastroesophageal cancers. Cancer.

[97] Lam R.C.T., Johnson D., Lam G., Li M.L.Y., Wong J.W.L., Lam W.K.J., Chan K.C.A., Ma B. (2022). Clinical applications of circulating tumor-derived DNA in the management of gastrointestinal cancers—Current evidence and future directions. Front. Oncol..

[98] Ghidini M., Hahne J.C., Senti C., Heide T., Proszek P.Z., Shaikh R., Carter P., Hubank M., Trevisani F., Garrone O. (2025). Circulating Tumor DNA Dynamics and Clinical Outcome in Metastatic Colorectal Cancer Patients Undergoing Front-Line Chemotherapy. Clin. Cancer Res..

[99] Huang A.H., Lee G.Y., Lu C.A., Sahin I.H., King G.T., Safyan R.A., Cohen S.A., Zhen D.B., Shankaran V., Harris W.P. (2025). Rechallenge with an Epidermal Growth Factor Receptor Inhibitor in Metastatic Colorectal Cancer: A Systematic Review and Meta-Analysis. JCO Oncol. Pract..

[100] Patelli G., Mauri G., Tosi F., Amatu A., Bencardino K., Bonazzina E., Pizzutilo E.G., Villa F., Calvanese G., Agostara A.G. (2023). Circulating Tumor DNA to Drive Treatment in Metastatic Colorectal Cancer. Clin. Cancer Res..

[101] Kuznetsova O., Battaiotto E., Malvezzi G., Gervaso L., Zampino M.G., Cella C.A., Benini L., Spada F., Fedyanin M., Tryakin A. (2026). Anti-EGFR rechallenge compared with standard of care for patients with ctDNA RAS/BRAF wild-type chemorefractory metastatic colorectal cancer: A systematic review and meta-analysis. Crit. Rev. Oncol. Hematol..

[102] Zhou J., Huang J., Zhou Z., Fan R., Deng X., Qiu M., Wu Q., Wang Z. (2025). Value of ctDNA in surveillance of adjuvant chemosensitivity and regimen adjustment in stage III colon cancer: A protocol for phase II multicentre randomised controlled trial (REVISE trial). BMJ Open.

[103] Zhou J., Zhang X., Liu Q., Li Y., Wu G., Fu W., Yao H., Wang Z., Xue H., Xu T. (2025). Rationale and design of a multicentre randomised controlled trial on circulating tumour DNA-guided neoadjuvant treatment strategy for locally advanced rectal cancer (CINTS-R). BMJ Open.

[104] Ciardiello D., Martini G., Boscolo Bielo L., Pietrantonio F., Raimondi A., Manca P., Pisconti S., Nisi C., Tortora G., Salvatore L. (2025). Cetuximab Rechallenge in Molecularly Selected Metastatic Colorectal Cancer: The Randomized CAVE-2 GOIM Trial. Ann. Oncol..

[105] Boige V., Bouché O., Mulot C., Evesque L., Ben Abdelghani M., Phelip J.M., Mineur L., Galais M.P., Villing A.L., Hautefeuille V. (2026). Circulating Tumor DNA Dynamics and Clinical Outcomes in Patients with Advanced Colorectal Cancer Treated with Cetuximab-Based Induction and Maintenance Treatment. Clin. Cancer Res..

[106] Misale S., Yaeger R., Hobor S., Scala E., Janakiraman M., Liska D., Valtorta E., Schiavo R., Buscarino M., Siravegna G. (2012). Emergence of KRAS mutations and acquired resistance to anti-EGFR therapy in colorectal cancer. Nature.

[107] Sartore-Bianchi A., Pietrantonio F., Lonardi S., Mussolin B., Rua F., Crisafulli G., Bartolini A., Fenocchio E., Amatu A., Manca P. (2022). Circulating tumor DNA to guide rechallenge with panitumumab in metastatic colorectal cancer: The phase 2 CHRONOS trial. Nat. Med..

[108] Lockwood C.M., Borsu L., Cankovic M., Earle J.S.L., Gocke C.D., Hameed M., Jordan D., Lopategui J.R., Pullambhatla M., Reuther J. (2023). Recommendations for Cell-Free DNA Assay Validations: A Joint Consensus Recommendation of the Association for Molecular Pathology and College of American Pathologists. J. Mol. Diagn..

[109] Chakravarty D., Johnson A., Sklar J., Lindeman N.I., Moore K., Ganesan S., Lovly C.M., Perlmutter J., Gray S.W., Hwang J. (2022). Somatic Genomic Testing in Patients with Metastatic or Advanced Cancer: ASCO Provisional Clinical Opinion. J. Clin. Oncol..

[110] Ji J., Wang C., Goel A., Melstrom K., Zerhouni Y., Lai L., Melstrom L., Raoof M., Fong Y., Kaiser A. (2024). Circulating Tumor DNA Testing in Curatively Resected Colorectal Cancer and Salvage Resection. JAMA Netw. Open.

[111] Reinert T., Petersen L.M.S., Henriksen T.V., Larsen M.Ø., Rasmussen M.H., Johansen A.F.B., Øgaard N., Knudsen M., Nordentoft I., Vang S. (2022). Circulating tumor DNA for prognosis assessment and postoperative management after curative-intent resection of colorectal liver metastases. Int. J. Cancer.

[112] Ryoo S.B., Heo S., Lim Y., Lee W., Cho S.H., Ahn J., Kang J.K., Kim S.Y., Kim H.P., Bang D. (2023). Personalised circulating tumour DNA assay with large-scale mutation coverage for sensitive minimal residual disease detection in colorectal cancer. Br. J. Cancer.

[113] Rubio-Alarcón C., Georgiadis A., Franken I.A., Wang H., van Nassau S.C.M.W., Schraa S.J., van der Kruijssen D.E.W., van Rooijen K., Linders T.C., Delis-van Diemen P. (2025). Circulating tumour DNA in patients with stage III colon cancer: Multicentre prospective PROVENC3 study. Br. J. Surg..

[114] Semenkovich N.P., Szymanski J.J., Earland N., Chauhan P.S., Pellini B., Chaudhuri A.A. (2023). Genomic approaches to cancer and minimal residual disease detection using circulating tumor DNA. J. Immunother. Cancer.

[115] Yang D., Yao Y., Gui F., Mei W., Zeng C. (2025). Decoding the epigenetic landscape: ctDNA methylation as a game-changer in hepatocellular carcinoma management. Biochim. Biophys. Acta Rev. Cancer.

[116] Zhou Q., Kang G., Jiang P., Qiao R., Lam W.K.J., Yu S.C.Y., Ma M.L., Ji L., Cheng S.H., Gai W. (2022). Epigenetic analysis of cell-free DNA by fragmentomic profiling. Proc. Natl. Acad. Sci. USA.

[117] Hu Q., Chen L., Li K., Liu R., Sun L., Han T. (2023). Circulating tumor DNA: Current implementation issues and future challenges for clinical utility. Clin. Chem. Lab. Med..

[118] Mi J., Wang R., Han X., Ma R., Li H. (2023). Circulating tumor DNA predicts recurrence and assesses prognosis in operable gastric cancer: A systematic review and meta-analysis. Medicine.

[119] Iden C.R., Mustafa S.M., Øgaard N., Henriksen T., Jensen S.Ø., Ahlborn L.B., Egebjerg K., Baeksgaard L., Garbyal R.S., Nedergaard M.K. (2025). Circulating tumor DNA predicts recurrence and survival in patients with resectable gastric and gastroesophageal junction cancer. Gastric Cancer.

[120] Liu Z., Wang G., Yang Y., Su Y., Zhang H., Liu J., Cui P., Fan X., Yang J., Zhang Z. (2025). ctDNA detects residual disease after neoadjuvant chemoradiotherapy and guides adjuvant therapy in esophageal squamous cell carcinoma. Cell Rep. Med..

[121] Sato S., Nakamura Y., Oki E., Yoshino T. (2023). Molecular Residual Disease-guided Adjuvant Treatment in Resected Colorectal Cancer: Focus on CIRCULATE-Japan. Clin. Color. Cancer.

