# Supplementary material for: Liquid Biopsy in Gastrointestinal Cancers: Circulating Tumor DNA for Molecular Residual Disease Assessment and Early Treatment Monitoring
Source: Cancers (Basel). 2026 Mar 20;18(6):1014. doi: 10.3390/cancers18061014 (PMC13025614; doi:10.3390/cancers18061014)
Supplement: Supplementary file 1 [file cancers-18-01014-s001.zip › cancers-4193795-supplementary.pdf]

Liquid Biopsy in Gastrointestinal Cancers: Circulating Tumor DNA for Molecular Residual Disease Assessment and Early Treatment Monitoring

Supplementary Digital File

CONTENT:

Supplementary Table S1. Full reproducible PubMed/MEDLINE search strategy 2
Supplementary Table S2. Representative quantitative anchors for ctDNA-based mRD assessment and monitoring across major gastrointestinal cancers 4
Supplementary Table S3: Additional studies and reporting framework 7
Supplementary Table S4: Clonal hematopoiesis (CH/CHIP) in ctDNA testing: recognition and mitigation of false positives 8
Supplementary Table S5: Pre-analytics and QC for ctDNA (MRD & monitoring): minimum standards, rejection rules, and reporting 9
Supplementary Table S6: Biological limitations of ctDNA: phenotype matrix and mitigation strategies 10
Supplementary Table S7: ctDNA monitoring: suggested schedules, trend interpretation, and clinical responses 11
Supplementary Table S8: Clinical map of ctDNA use in gastrointestinal cancers: maturity, actionability, and limitations 13
Supplementary Table S9. Landmark published and ongoing ctDNA studies across major gastrointestinal cancers 15

Supplementary Table S1. Full reproducible PubMed/MEDLINE search strategy

Line	Search statement
**1**	(<br>"Circulating Tumor DNA"[MeSH] OR "Cell-Free Nucleic Acids"[MeSH] OR "Liquid Biopsy"[MeSH]<br>OR "circulating tumor DNA"[tiab] OR "circulating tumour DNA"[tiab] OR ctDNA[tiab]<br>OR "cell-free DNA"[tiab] OR "cell free DNA"[tiab] OR cfDNA[tiab]<br>OR "circulating cell-free DNA"[tiab] OR "liquid biopsy"[tiab]<br>)
**2**	(<br>"Digestive System Neoplasms"[MeSH] OR "Gastrointestinal Neoplasms"[MeSH] OR "Biliary Tract Neoplasms"[MeSH]<br>OR "Carcinoma, Hepatocellular"[MeSH] OR colorectal[tiab] OR "colorectal cancer"[tiab] OR "colon cancer"[tiab]<br>OR "rectal cancer"[tiab] OR gastric[tiab] OR "gastric cancer"[tiab] OR gastroesophageal[tiab]<br>OR "gastro-oesophageal"[tiab] OR esophageal[tiab] OR oesophageal[tiab] OR pancreatic[tiab]<br>OR "pancreatic cancer"[tiab] OR PDAC[tiab] OR cholangiocarcinoma[tiab] OR "biliary tract"[tiab]<br>OR "hepatocellular carcinoma"[tiab] OR HCC[tiab]<br>)
**3**	(<br>"molecular residual disease"[tiab] OR "minimal residual disease"[tiab] OR MRD[tiab]<br>OR surveillance[tiab] OR "follow-up"[tiab] OR recurrence[tiab] OR relapse[tiab]<br>OR postoperative[tiab] OR "post-operative"[tiab] OR postresection[tiab] OR "post-resection"[tiab]<br>OR adjuvant[tiab] OR perioperative[tiab] OR neoadjuvant[tiab]<br>)
**4**	(<br>monitor*[tiab] OR kinetics[tiab] OR dynamic*[tiab] OR serial[tiab] OR longitudinal[tiab]<br>OR response[tiab] OR progression[tiab] OR "treatment monitoring"[tiab] OR "early response"[tiab]<br>)
**5**	(<br>resistance[tiab] OR resistant[tiab] OR rechallenge[tiab] OR "treatment selection"[tiab]<br>OR clonal[tiab] OR "acquired resistance"[tiab] OR "anti-EGFR"[tiab]<br>OR EGFR[tiab] OR RAS[tiab] OR BRAF[tiab]<br>)
**6**	(<br>"error suppression"[tiab] OR "error-suppression"[tiab] OR "error corrected"[tiab] OR "error-corrected"[tiab]<br>OR "error correction"[tiab] OR "unique molecular identifier"[tiab] OR "unique molecular identifiers"[tiab]<br>OR UMI[tiab] OR duplex[tiab] OR "duplex sequencing"[tiab] OR "clonal hematopoiesis"[tiab] OR CHIP[tiab]<br>OR preanalytic*[tiab] OR "pre-analytic"[tiab] OR "pre-analytical"[tiab]<br>OR "limit of detection"[tiab] OR LoD[tiab] OR LoQ[tiab] OR methylation[tiab] OR fragmentom*[tiab]<br>)

**7**	1 AND 2 AND 3
**8**	1 AND 2 AND 4
**9**	1 AND 2 AND 5
**10**	1 AND 2 AND 6
**11**	7 OR 8 OR 9 OR 10
**12**	11 AND english[lang] NOT ("Animals"[MeSH] NOT "Humans"[MeSH])

Supplementary Table S2. Representative quantitative anchors for ctDNA-based mRD assessment and monitoring across major gastrointestinal cancers

Tumor type / clinical setting	Representative study	Assay / window	Key quantitative anchor(s)	What the number means clinically	Key caveat
CRC, post-operative mRD after curative-intent resection	Faulkner et al. (systematic review and meta-analysis) [1]	Mixed assays; first post-operative liquid biopsy	PFS HR 6.92 (95% CI 4.49–10.64) for ctDNA-positive vs ctDNA-negative patients	Post-op ctDNA positivity is not a subtle signal in CRC; it marks a major increase in recurrence risk	Cross-study heterogeneity remained substantial; timing and assay class differed across included studies
CRC, stage II colon cancer, ctDNA-guided adjuvant strategy	DYNAMIC trial, 5-year outcomes [2,3]	Tumor-informed ctDNA; post-op weeks 4 and 7	2-year RFS 93.5% vs 92.4% (ctDNA-guided vs standard management), with reduced chemotherapy exposure	The strongest strategy-level evidence in GI oncology: less adjuvant chemotherapy without compromising oncologic outcomes	Applies most directly to stage II colon cancer; should not be extrapolated wholesale to all CRC settings
Gastric / gastroesophageal adenocarcinoma, post-operative / perioperative MRD	Meta-analysis of 8 studies [4,5]	Mixed assays; perioperative/post-operative timepoints	Post-operative ctDNA associated with recurrence risk RR 3.17–3.68; pooled RFS HR 6.37 (95% CI 2.70–15.01); post-operative timing showed highest prognostic value (RR 14.09, 95% CI 7.31–27.15)	Quantitative support that post-op ctDNA in upper GI disease is clinically meaningful, especially after surgery	Assays and sampling windows were heterogeneous; negative ctDNA remains less reassuring than in CRC
PDAC, post-op and surveillance after resection	Guo et al. [6]	Post-op window vs adjuvant/surveillance window	Post-op: sensitivity 35.7%, specificity 88.9%, PPV 79.6%, NPV 53.2%. Adjuvant/surveillance window: sensitivity	This row quantifies the central PDAC asymmetry: a positive result is highly informative, whereas a negative result is often	Single-institution retrospective study; low shedding remains a structural limitation

			62.5%, specificity 95.5%, PPV 94.4%, NPV 67.7%	not sufficient to exclude residual disease	
Extrahepatic cholangiocarcinoma, resected, during adjuvant therapy	Yoo et al. (STAMP extended follow-up) [7]	Tumor-informed ctDNA before and during ACT	ctDNA positivity associated with inferior DFS: HR 1.8 before ACT, HR 7.72 at 12 weeks on ACT, HR 5.24 at 24 weeks on ACT, HR 3.81 anytime post-surgery. Serially ctDNA-negative patients had longer DFS than sustained positives (HR 6.7) or converters (HR 5.8)	Strong quantitative support that serial ctDNA dynamics during ACT carry more information than a single static postoperative measurement	Data are strongest for resected extrahepatic cholangiocarcinoma, not all BTC subtypes uniformly
HCC, non-metastatic disease after curative-intent treatment	Buonopane et al. (systematic review and meta-analysis) [8]	Plasma ctDNA after curative-intent treatment	Postoperative ctDNA positivity associated with shorter RFS HR 4.48 (95% CI 2.56–7.82) and worse OS HR 2.99 (95% CI 1.94–4.61); reported sensitivity ranged 33–82% and specificity 41–100%	Good example of “prognostically strong but methodologically heterogeneous”: signal is real, but assay variability is substantial	Retrospective evidence base; wide performance ranges reflect assay heterogeneity and cirrhosis-related background cfDNA

Footnote: Quantitative results are shown as representative anchors from pivotal studies or meta-analyses and should not be interpreted as directly comparable pooled estimates across tumor types, because assay class, sampling windows, positivity definitions, and endpoints differ substantially.

Abbreviations: ACT, adjuvant chemotherapy; BTC, biliary tract cancer; CI, confidence interval; CRC, colorectal cancer; ctDNA, circulating tumor DNA; DFS, disease-free survival; HCC, hepatocellular carcinoma; HR, hazard ratio; mRD, molecular residual disease; NPV, negative predictive value; OS, overall survival; PDAC, pancreatic ductal adenocarcinoma; PFS, progression-free survival; PPV, positive predictive value; RFS, recurrence-free survival; RR, relative risk.

References:

1. Faulkner, L.G.; Howells, L.M.; Pepper, C.; Shaw, J.A.; Thomas, A.L. The utility of ctDNA in detecting minimal residual disease following curative surgery in colorectal cancer: A systematic review and meta-analysis. Br. J. Cancer 2023, 128, 297–309. https://doi.org/10.1038/s41416-022-02017-9.
2. Tie, J.; Cohen, J.D.; Lahouel, K.; Lo, S.N.; Wang, Y.; Wong, R.; Shapiro, J.D.; Harris, S.J.; Khattak, A.; Burge, M.E.; et al. Circulating tumor DNA analysis guiding adjuvant therapy in stage II colon cancer. N. Engl. J. Med. 2022, 386, 2261–2272. https://doi.org/10.1056/NEJMoa2200075.
3. Tie, J.; Wang, Y.; Lo, S.N.; Lahouel, K.; Cohen, J.D.; Wong, R.; Shapiro, J.D.; Harris, S.J.; Khattak, A.; Burge, M.E.; et al. Circulating tumor DNA analysis guiding adjuvant therapy in stage II colon cancer: 5-year outcomes of the randomized DYNAMIC trial. Nat. Med. 2025, 31, 1509–1518. https://doi.org/10.1038/s41591-025-03579-w.
4. Mi, J.; Wang, R.; Han, X.; Ma, R.; Li, H. Circulating tumor DNA predicts recurrence and assesses prognosis in operable gastric cancer: A systematic review and meta-analysis. Medicine (Baltimore) 2023, 102, e36228. https://doi.org/10.1097/MD.0000000000036228.
5. Iden, C.R.; Mustafa, S.M.; Øgaard, N.; Henriksen, T.; Jensen, S.Ø.; Ahlborn, L.B.; Egebjerg, K.; Baeksgaard, L.; Garbyal, R.S.; Nedergaard, M.K.; et al. Circulating tumor DNA predicts recurrence and survival in patients with resectable gastric and gastroesophageal junction cancer. Gastric Cancer 2025, 28, 83–95. https://doi.org/10.1007/s10120-024-01556-9.
6. Guo, M.Z.; Sachidanand, A.S.; Nguyen, T.; Patel, S.D.; Burns, W.R.; Burkhart, R.; Le, D.T.; Lafaro, K.; Bever, K.M.; Pishvaian, M.J.; et al. Postsurgical circulating tumor DNA as a prognostic biomarker for relapse of resected pancreatic ductal adenocarcinoma. J. Gastrointest. Surg. 2026, 30, 102359. https://doi.org/10.1016/j.gassur.2026.102359.
7. Yoo, C.; Jeong, H.; Jeong, J.H.; Kim, K.-P.; Lee, S.; Ryoo, B.-Y.; Hwang, D.W.; Lee, J.H.; Moon, D.-B.; Kim, K.-H.; et al. Circulating tumor DNA status and dynamics predict recurrence in patients with resected extrahepatic cholangiocarcinoma. J. Hepatol. 2025, 82, 861–870. https://doi.org/10.1016/j.jhep.2024.10.043.
8. Buonopane, I.R.; Saldanha, E.F.; de Menezes, J.S.A.; da Conceição, L.D.; Reis, C.M.P.; Leite, L.F.; Francischetto, T.; D'Alpino Peixoto, R.; de Castria, T.B. Circulating tumour DNA for a minimal residual disease assessment and recurrence risk in hepatocellular carcinoma: A systematic review and meta-analysis. Br. J. Cancer 2026, 134, 699–706. https://doi.org/10.1038/s41416-025-03296-8.

Supplementary Table S3: Additional studies and reporting framework

Evidence domain (what the study claims)	Typical study types to include (fill with your list)	Why it belongs in Supplementary	Minimum reporting fields to ensure comparability
Post-operative MRD (smaller cohorts / subgroup analyses)	Smaller prospective cohorts; retrospective validations; subgroup analyses (e.g., MSI status, tumor sidedness, stage strata)	High heterogeneity in assays and sampling windows; too detailed for main text	Sampling window (days post-op; off-window handling); assay class (TI/TA/signature); positivity definition (incl. borderline/indeterminate); number of tracked markers (if TI) or panel breadth (if TA); LOD/LoQ; plasma volume; follow-up duration and recurrence definition.
Surveillance and lead time	Serial sampling cohorts; "intensified" vs standard follow-up observational comparisons	Different schedules/endpoints create bias; action after ctDNA+ often unreported	Sampling schedule and adherence; pre-specified imaging trigger after ctDNA+; definition of lead time; handling of ctDNA+/imaging−; FP/FN definitions; censoring rules; how interval cancers were counted.
Metastatic CRC: response kinetics	Observational studies linking early ctDNA change to response/PFS/OS	Frequently non-randomized; endpoints and timepoints vary	Baseline ctDNA detectability/TF; exact on-treatment timepoints (e.g., week 2–4); definition of "response" in ctDNA (percent drop, absolute change, clearance); correlation with RECIST timing; missing data handling; treatment line and regimen.
Resistance and treatment selection (advanced disease)	Studies on resistance alterations, clonal dynamics, rechallenge selection	Platform-dependent signals; CHIP confounding for TA mutation panels	Genes/alteration classes tracked; panel breadth; LOD and low-VAF policy; CHIP mitigation strategy; pre-specified decision thresholds; confirmation rules (repeat sampling); linkage to clinical decision points and imaging.
Methylation / fragmentomics (epigenetic signatures)	Early validations; model development/transfer studies; GI-specific performance reports	Model-specific and often non-transferable; inappropriate to summarize in main	Training vs validation cohorts (separated); intended use (screening vs MRD vs monitoring); locked cut-offs; AUC/sensitivity/specificity with CI; pre-analytic requirements; performance in the same clinical window; drift/transportability checks.
Pre-analytics / analytical method papers	SOP comparisons; tube/processing studies; error-suppression method reports	Technical granularity; critical for interpreting negative results	Tube type; time-to-spin; centrifugation protocol; plasma volume; storage/freeze–thaw; library prep/UMI/duplex method; run-level QC metrics; failure rates; sample exclusion rules.

Abbreviations: AUC, area under the curve; CI, confidence interval; CRC, colorectal cancer; ctDNA, circulating tumor DNA; FP/FN, false positive/false negative; LOD/LoD, limit of detection; LoQ, limit of quantification; MRD/mRD, molecular residual disease; MSI, microsatellite instability; OS, overall survival; PFS, progression-free survival; RECIST, Response Evaluation Criteria in Solid Tumors; TA, tumor-agnostic; TF, tumor fraction; TI, tumor-informed; UMI, unique molecular identifier.

Supplementary Table S4: Clonal hematopoiesis (CH/CHIP) in ctDNA testing: recognition and mitigation of false positives

High-risk context for CH/CHIP	Red flags in the ctDNA report	Minimum mitigation (lab/bioinformatics)	Safest clinical handling + what to report in the manuscript
Older age (e.g., >60), prior hematologic disorder, chronic inflammation, smoking history	Single low-VAF SNV with no supporting tumor signal; variant appears in canonical CHIP genes; discordance with clinical picture	MUST: CHIP-aware filtering (gene lists + contextual rules). Prefer matched WBC sequencing when feasible. Use error suppression (UMI/duplex) and contamination controls.	Clinical: Treat as possible CHIP until proven otherwise; repeat plasma (2–4 weeks if feasible) and seek orthogonal confirmation (tumor tissue/archival, imaging, MDT) before any escalation. Report: whether WBC-matched was used; CHIP filtering rules; proportion flagged/excluded as CHIP.
Tumor-agnostic (TA) mutation panels used in MRD	"MRD+" driven by a single variant, especially in CHIP-enriched genes; low TF setting; no multi-marker support	MUST: Pre-specified positivity criteria (e.g., ≥2 independent tumor markers or serial confirmation). Exclude/label likely CHIP variants from MRD aggregation.	Clinical: Do not trigger adjuvant intensification on single-marker, low-VAF TA positives. Require confirmation (repeat/serial trend) and/or orthogonal evidence. Report: MRD positivity definition; how CHIP genes were handled; indeterminate category policy.
Borderline positive result (near LOD) on a single draw	VAF near LOD; inconsistent across replicates; "one-off" positivity without trend	MUST: Library or technical replicates for borderline calls; sample-level LOD/LoQ reporting; strict run controls; contamination checks	Clinical: Label as indeterminate; repeat sampling (2–4 weeks when clinically acceptable) before acting. Report: indeterminate definition; repeat-testing plan; sample-level QC/LOD reporting.
Post-chemotherapy or post-radiotherapy	Multiple low-VAF variants; atypical spectra; signal not coherent with known tumor biology	MUST: Negative controls, artifact modeling, and preferably WBC-matched to separate therapy-related hematopoietic clones from tumor signal	Clinical: Interpret low-level multi-variant findings cautiously; integrate with imaging and clinical course; avoid reflex escalation. Report: timing relative to systemic therapy; how therapy-associated artifacts were controlled.
Low ctDNA detectability / ultra-low TF (common in MRD)	"Positive" call based on minimal molecules; unstable detection between timepoints	MUST: Explicit reporting of plasma volume and molecule counts where available; conservative thresholds; requirement for serial confirmation	Clinical: For high-stakes decisions, require two consecutive positives or rising trend; if discordant, rely on imaging/MDT. Report: plasma volume, assay sensitivity, and serial confirmation rules.
Discordant biology: ctDNA positive but imaging negative	Low-level "MRD+" driven by a CHIP-suspect variant; no trend	MUST: Ensure CHIP filtering + repeat sample; consider orthogonal assay class (e.g., TI vs TA where appropriate)	Clinical: Follow a predefined "ctDNA+/imaging–" pathway (repeat ctDNA + scheduled imaging) rather than ad hoc therapy. Report: your algorithm for discordant results and how CHIP was excluded.

Abbreviations: CH, clonal hematopoiesis; CHIP, clonal hematopoiesis of indeterminate potential; ctDNA, circulating tumor DNA; MRD/mRD, molecular residual disease; SNV, single nucleotide variant; TA, tumor-agnostic; TF, tumor fraction; UMI, unique molecular identifier; VAF, variant allele fraction; WBC, white blood cells; LOD/LoD, limit of detection; LoQ, limit of quantification; MDT, multidisciplinary team.

Supplementary Table S5: Pre-analytics and QC for ctDNA (MRD & monitoring): minimum standards, rejection rules, and reporting

Process step	Main risk	Bias on ctDNA result	Priority + minimum standard (incl. "if–then" rule) + what to report
Tube type	EDTA used with delayed processing; mixed tube types	↑ background cfDNA → MRD false negatives	MUST: Harmonize tube type across sites. If processing cannot be rapid, use cfDNA-stabilizing tubes; if EDTA, process rapidly. Report: tube type/manufacturer; harmonization plan.
Time-to-spin	Delays/variability between samples	↓ TF, ↑ noise → false negatives; poor longitudinal comparability	MUST: Pre-specify allowable window; flag samples outside window; consider repeat draw if clinically feasible. Report: median (IQR) time-to-spin; % outside limit; handling of outliers (exclude/flag).
Plasma separation	Residual cells/platelets; inconsistent centrifugation	gDNA contamination → noise; borderline calls become unreliable	MUST: Two-step spin for ctDNA plasma. If single-spin used, flag for cautious interpretation (especially low-level positives/negatives). Report: g-force/time/temp; SOP; failure rate.
Plasma volume	Low/variable volume	↑ effective LOD → false negatives (dominant in MRD)	MUST: Use maximum feasible volume and keep consistent within patient. If volume below assay minimum, do not interpret a negative as rule-out. Report: mL processed; cfDNA yield (ng and ng/mL).
Hemolysis/lipemia/clots	Inhibitors or poor extraction	Failed runs or false negatives	MUST: Pre-specify rejection criteria; repeat sampling when possible. Report: rejection rate and reasons; any hemolysis scoring used.
Storage & freeze–thaw	Variable storage; repeated freeze–thaw	Degradation/batch effects → spurious "clearance"/fluctuations	MUST: Aliquot; −80°C; minimize freeze–thaw. If multiple freeze–thaw cycles occur, flag longitudinal comparisons. Report: storage duration/temp; freeze–thaw count; batch handling strategy.
Post-op MRD window	Sampling too early post-op; inconsistent timing	Trauma cfDNA surge → false negatives; transient signals	MUST: Pre-specify post-op window; treat off-window samples as non-comparable unless justified. Report: day post-op (median/IQR); % outside window; analytic handling of off-window samples.
Serial sampling	Irregular intervals; assay/platform changes	Trend uninterpretable; artificial shifts	MUST: Fixed schedule + same platform for longitudinal decisions. If platform changes, do not compare quantitatively without bridging. Report: schedule adherence; platform/version changes; bridging approach.
Analytical QC in report	No borderline policy; missing LOD/LoQ; no sample-level QC flags	Over-/under-calling low-level disease	MUST: Define positivity threshold and borderline zone with repeat-testing policy. Report: LOD/LoQ; positivity criteria; replicate requirements; sample-level QC pass/fail.

Abbreviations: ACT, adjuvant chemotherapy; cfDNA, cell-free DNA; ctDNA, circulating tumor DNA; gDNA, genomic DNA; IFU, instructions for use (manufacturer's instructions); IQR, interquartile range; LOD/LoD, limit of detection; LoQ, limit of quantification; MRD/mRD, (molecular) residual disease / molecular residual disease; MDT, multidisciplinary team; QC, quality control; SOP, standard operating procedure; TF, tumor fraction; UMI, unique molecular identifier; VAF, variant allele fraction.

Supplementary Table S6: Biological limitations of ctDNA: phenotype matrix and mitigation strategies

**Phenotype / clinical context**	**Expected ctDNA behavior**	**Main risk (FN/FP)**	**How to recognize it (practical clues)**	**Mitigation (minimum harm strategy)**
Peritoneal-only metastases / peritoneal carcinomatosis	Low shedding into blood; plasma ctDNA may remain negative despite active disease	High FN	Imaging or symptoms consistent with peritoneal involvement; mucinous features; discordant clinical course	Do not use ctDNA as rule-out. Prioritize site-directed imaging and MDT review. Consider alternative compartments (e.g., ascites) where clinically appropriate. Use serial assessment rather than single timepoints.
Mucinous histology (CRC and others)	Reduced plasma detectability; ctDNA often lower than expected for disease burden	High FN	Pathology report: mucinous component; frequent peritoneal pattern; low baseline ctDNA	Increase plasma volume where feasible; choose assays optimized for low TF; rely on trend and imaging. Treat negative MRD as conditional.
Very low tumor burden / oligometastatic disease	Ultra-low tumor fraction close to LOD; intermittent detectability	High FN	Minimal radiologic disease; low/normal tumor markers; weak/unstable ctDNA signal	Maximize plasma volume; avoid overinterpreting single negatives; confirm with serial sampling and imaging. Consider assay choice (lower LOD, TI where feasible).
Early post-operative or post-procedural period	High background cfDNA from tissue injury dilutes tumor fraction	FN + high variability	Sampling within an early post-op window; high inflammatory markers; inconsistent results across early draws	Pre-specify the post-op MRD window and adhere to it. Flag off-window samples as non-comparable. Avoid declaring “clearance” based on early post-op negatives.
Cirrhosis / active hepatitis (HCC)	High cfDNA background; epigenetic signals may vary with inflammation	FP/FN	Known cirrhosis, hepatitis flares, elevated transaminases; variable AFP	Prefer trend-based interpretation. Correlate with AFP and imaging. Avoid acting on isolated low-level positives/negatives during inflammatory flares; repeat when clinically stable.
Cholangitis or biliary infection (BTC/CCA)	Increased cfDNA background and assay noise	FP/FN	Fever/CRP elevation, cholestasis, biliary obstruction, recent instrumentation	Avoid ctDNA draws during acute infection. Repeat after clinical resolution. Interpret borderline positives cautiously; confirm with imaging and clinical context.

Abbreviations: AFP, alpha-fetoprotein; BTC, biliary tract cancers; CCA, cholangiocarcinoma; CRC, colorectal cancer; ctDNA, circulating tumor DNA; cfDNA, cell-free DNA; FN, false negative; FP, false positive; HCC, hepatocellular carcinoma; LOD, limit of detection; MDT, multidisciplinary team; MRD/mRD, molecular residual disease; TF, tumor fraction.

Supplementary Table S7: ctDNA monitoring: suggested schedules, trend interpretation, and clinical responses

Setting	Practical prerequisites (when ctDNA is useful)	Suggested sampling schedule (example)	Trend interpretation → recommended clinical response (with pitfalls)
Resected CRC (surveillance after curative intent)	Assay validated for MRD; consistent platform over time; pre-defined pathway for ctDNA+/imaging−	Example: every 3 months for 2 years, then every 6 months to year 5 (align with routine follow-up visits)	New positivity or rising trend → repeat ctDNA in a short interval (e.g., 2–4 weeks) if low-level/borderline; initiate site-directed imaging and MDT review. Pitfalls: single borderline call; pre-analytic variability; low-shedding/peritoneal disease; interpreting a negative as universal rule-out.
Metastatic CRC on systemic therapy	Baseline ctDNA detectable (document TF/LOD); stable pre-analytics; same assay for longitudinal comparisons	Baseline; 2–4 weeks (early kinetics); then aligned with standard imaging cadence (e.g., every 8–12 weeks)	No decline / sustained rise can precede radiology but is not RECIST → consider earlier imaging if discordant with symptoms/biochemistry; confirm adherence and rule out sampling/QC artefacts. Pitfalls: overreacting to one data point; TF fluctuations; transient changes around treatment interruption ("flare"); mixed/oligoprogressive patterns.
Anti-EGFR rechallenge (mCRC)	Prior clinical benefit from anti-EGFR and a drug holiday; ctDNA platform capable of tracking resistance categories; baseline detectability confirmed	Immediately pre-rechallenge; optional on-treatment sampling if an early reassessment plan exists; repeat at clinical concern	No resistance signal above LOD supports proceeding with rechallenge (biomarker-enriched selection). Rising resistance signal → bring imaging forward and plan next line (do not switch on ctDNA alone).

			Pitfalls: narrow panels; low TF leading to false reassurance; inconsistent thresholds; lack of a predefined action plan for borderline results.
PDAC / BTC monitoring (advanced disease)	ctDNA detectable at baseline or a validated signature assay available; recognize low-shedding biology; avoid “ctDNA-only” decisions	Baseline; then every 4–8 weeks depending on regimen and clinical tempo (often aligned to visits)	Rapid upward trend supports early reassessment and consideration of resistance/targets; confirm with CT/MRI and clinical context. Pitfalls: inflammation/cholangitis increasing background cfDNA; intermittent detectability; low shedding; assay transfer from screening to on-treatment settings without validation.

Abbreviations: BTC, biliary tract cancers; CRC, colorectal cancer; ctDNA, circulating tumor DNA; LOD, limit of detection; MRD/mRD, molecular residual disease; MDT, multidisciplinary team; PDAC, pancreatic ductal adenocarcinoma; RECIST, Response Evaluation Criteria in Solid Tumors; TF, tumor fraction.

Supplementary Table S8: Clinical map of ctDNA use in gastrointestinal cancers: maturity, actionability, and limitations

Tumor type	Clinical scenario	Most useful ctDNA signal (fit-for-purpose)	Evidence maturity / actionability	Most defensible interpretation	Main biological limitation(s)
CRC	Post-operative MRD (stage II–III)	TI multi-variant MRD; serial status and clearance	Routine (highest maturity in GI)	Rule-in: MRD+ identifies very high relapse risk. Rule-out: MRD− is conditional (assay/timing/shedding).	Low shedding in mucinous/peritoneal disease; post-op timing window; ultra-low TF near LOD.
CRC	Surveillance after curative-intent therapy	Serial ctDNA (trend) ± TI MRD	Selective (algorithm-dependent)	Early molecular relapse signal; requires a predefined imaging pathway to avoid ad hoc escalation.	Lead time can drive overtesting; false alarms (borderline calls); variable ctDNA detectability.
CRC (metastatic)	Anti-EGFR resistance and rechallenge	TA resistance categories (RAS/BRAF/EGFR-ECD) and clone decay	Selective (strongest for rechallenge selection)	Rule-in: supports patient selection for rechallenge and earlier confirmation of resistance. Does not replace RECIST.	Variable TF; clonal heterogeneity; "negative resistance" may reflect low TF/panel limits.
Gastric / GEJ	Monitoring systemic therapy	TA mutations and/or Sig (platform-dependent)	Selective → Trial-preferred (heterogeneous data)	Best used as supportive trend alongside imaging and symptoms; not a stand-alone trigger for switching therapy.	Often low shedding; biologic heterogeneity; peritoneal disease under-represented in plasma.
Gastric / GEJ	Post-gastrectomy MRD	TI MRD if baseline detectable	Trial-preferred	Rule-in: a positive MRD is concerning and should prompt careful reassessment; rule-out is weak vs CRC.	Low shedding; peritoneal micrometastases; post-op background cfDNA.
Esophageal / GEJ	After chemoradiation and/or surgery	Serial ctDNA ± Sig in selected platforms	Trial-preferred	May precede radiology; use only with a protocolized confirmation strategy (repeat + imaging plan).	Treatment-related inflammation and cfDNA background; variable shedding; timing sensitivity.

PDAC	Monitoring systemic therapy	Sig and/or KRAS tracking when measurable	Selective → Trial-preferred	Supportive trend may help timing of imaging and clinical reassessment; not a substitute for CT.	Very low shedding in a subset; inflammatory background; intermittent detectability.
PDAC	Post-resection MRD	TI MRD if baseline detectable	Trial-preferred	Rule-in: positive MRD is strongly adverse; rule-out limited due to detectability constraints.	Low baseline ctDNA detectability; rapid kinetics; post-op background cfDNA.
Biliary tract cancers (BTC/CCA)	Target identification & resistance	TA panels for actionable alterations (e.g., FGFR2/IDH1 categories)	Routine for genotyping in advanced disease; monitoring = Selective	Genotyping is clinically useful; ctDNA can support resistance monitoring when ctDNA is measurable.	Cholangitis/inflammation elevates cfDNA background; low TF; biliary obstruction effects.
HCC	Monitoring after locoregional therapy / transplant setting	Sig (methylation/fragmentomics) ± mutations (platform-dependent)	Trial-preferred → Selective (center-dependent)	Trend may be useful, but interpret cautiously due to high background; confirmation with imaging/AFP often needed.	Cirrhosis elevates cfDNA background → false positives and reduced specificity; variable shedding.

Abbreviations: AFP, alpha-fetoprotein; BTC, biliary tract cancers; CCA, cholangiocarcinoma; CRC, colorectal cancer; ctDNA, circulating tumor DNA; cfDNA, cell-free DNA; GEJ, gastroesophageal junction; HCC, hepatocellular carcinoma; LOD, limit of detection; MRD/mRD, molecular residual disease; PDAC, pancreatic ductal adenocarcinoma; RECIST, Response Evaluation Criteria in Solid Tumors; Sig, methylation/fragmentomics signatures; TA, tumor-agnostic (mutation panels); TF, tumor fraction; TI, tumor-informed.

Supplementary Table S9. Landmark published and ongoing ctDNA studies across major gastrointestinal cancers

**Panel A. Published landmark studies**				
**Tumor type**	**Key study (design; N)**	**ctDNA approach**	**Clinical setting**	**Main result / why it matters**
CRC (colon)	DYNAMIC (randomized trial; n = 455) [1,2]	Tumor-informed mutation-based assay; post-op weeks 4 and 7	Stage II colon cancer; ctDNA-guided adjuvant strategy	ctDNA-guided management reduced adjuvant chemotherapy use (15% vs 28%) without compromising recurrence-free survival (2-year RFS 93.5% vs 92.4%; 5-year RFS 88% vs 87%). This remains the clearest strategy-level ctDNA evidence in GI oncology.
CRC (resectable)	GALAXY / CIRCULATE-Japan (prospective multicenter observational study; n = 2,240) [3]	Personalized tumor-informed 16-plex PCR-NGS MRD assay	Post-surgical MRD window and surveillance after curative-intent surgery	ctDNA positivity during the MRD window was strongly associated with inferior outcomes (DFS HR 11.99; OS HR 9.68). Clearance on adjuvant therapy identified a more favorable subgroup. This is the largest prospective postoperative CRC ctDNA cohort.
Rectal cancer	Ando et al. (prospective cohort; n = 250) [4]	Personalized tumor-informed 16-plex PCR-NGS assay	Stage II–III rectal cancer treated with upfront surgery	Postoperative ctDNA status and serial dynamics predicted recurrence risk; patients with sustained positivity or conversion from negative to positive had the worst DFS. The study also supports a potential interaction between ctDNA positivity and adjuvant chemotherapy benefit.
mCRC	CHRONOS (phase II, single-arm interventional trial; 52 screened / 27 treated) [5]	ctDNA ddPCR screening for RAS/BRAF/EGFR-ECD resistance alterations	Later-line anti-EGFR rechallenge	Of screened patients, 31% were molecularly excluded. Among treated patients, ORR was 30% and disease control 63%. This is the clearest proof-of-concept that ctDNA can directly drive treatment selection in metastatic CRC.

Gastric / GEJ adenocarcinoma	Iden et al. (prospective cohort; 86 patients; 229 plasma samples) [6]	Methylation-based ddPCR assay (TriMeth) targeting C9orf50, KCNQ5, CLIP4	Perioperative chemotherapy and surgery	ctDNA after one cycle of chemotherapy was associated with worse RFS (HR 2.54) and OS (HR 2.23); detectability fell from 56% at baseline to 15% postoperatively. This supports perioperative dynamics as a biologically meaningful signal in upper GI disease.
Esophageal squamous cell carcinoma	Liu et al. (prospective cohort; n = 132) [7]	Personalized tumor-informed ctDNA assay plus fixed-panel comparator	After neoadjuvant chemoradiotherapy (nCRT) and post-surgery	Personalized ctDNA outperformed fixed panels for MRD detection. Adding ctDNA to conventional clinical assessment increased sensitivity for identifying non-pCR to 92.0–93.2%, and post-surgical MRD identified patients more likely to benefit from adjuvant immunotherapy.
Metastatic gastroesophageal cancer	Tatalovic et al. (prospective serial monitoring cohort; n = 37) [8]	Tissue-informed ddPCR with serial ctDNA kinetics	Early on-treatment monitoring during systemic therapy	A decline in ctDNA to <57.1% of baseline after 2 weeks predicted later CT-based response with 90% specificity and stratified both OS and PFS. This is a representative "early kinetics" study rather than a strategy trial.
PDAC	Guo et al. (post-surgical cohort; n = 51) [9]	Postoperative mutation-based ctDNA testing	After curative-intent resection	Postoperative ctDNA had sensitivity 35.7%, specificity 88.9%, PPV 79.6%, and NPV 53.2% for relapse prediction. This quantitatively illustrates the PDAC asymmetry: a positive result is informative, whereas a negative result is often not.
Extrahepatic cholangiocarcinoma	Yoo et al. / STAMP biomarker cohort (101 randomized in parent ACT trial; 89 in biomarker cohort) [10]	Personalized tumor-informed 16-plex PCR-NGS assay	Post-surgery and during adjuvant chemotherapy	ctDNA positivity was associated with inferior DFS before ACT (HR 1.8), at 12 weeks (HR 7.72), at 24 weeks (HR 5.24), and anytime post-surgery (HR 3.81). Serially ctDNA-negative patients had the best DFS.
HCC	Buonopane et al. (systematic review/meta-	Mixed plasma ctDNA assays	Non-metastatic HCC after curative-intent treatment	Postoperative ctDNA positivity was associated with shorter RFS (HR 4.48) and worse OS (HR 2.99). This is currently

	analysis; 10 studies, n = 793) [11]			among the best quantitative summaries in HCC, but no ctDNA-guided interventional trial has yet established practice-changing utility.
**Panel B. Ongoing ctDNA-guided interventional trials / platforms**				
**Tumor type**	**Trial / status**	**ctDNA role**	**Population / setting**	**Main strategic question**
CRC (colon)	CIRCULATE-US / NRG-GI008 (phase II/III randomized platform; ongoing) [12,13]	Postoperative ctDNA used for de-escalation if negative and escalation if positive	Resected early-stage colon cancer	Can postoperative ctDNA safely reduce adjuvant treatment in low-risk patients and intensify therapy in molecularly positive patients?
CRC (colon)	VEGA / ALTAIR within CIRCULATE-Japan (phase III platform) [14,15]	ctDNA-negative patients enter de-escalation; ctDNA-positive patients enter escalation strategy	High-risk stage II / low-risk stage III colon cancer and molecularly positive post-op patients	Can ctDNA support both omission of chemotherapy in MRD-negative disease and pre-emptive treatment in MRD-positive disease?
Stage III colon cancer	REVISE (phase II randomized protocol; planned n = 60) [16]	Persistent ctDNA positivity after two XELOX cycles triggers treatment intensification	Stage III colon cancer during adjuvant therapy	Does switching persistent ctDNA-positive patients from XELOX to FOLFOXIRI improve ctDNA clearance and 2-year DFS?
Rectal cancer	CINTS-R (multicenter open-label randomized protocol; planned n = 470) [17]	Baseline and early-treatment ctDNA incorporated into neoadjuvant treatment assignment	Locally advanced rectal cancer	Can ctDNA guide choice/intensity of neoadjuvant therapy more effectively than conventional strategy alone?
Upper GI / PDAC / BTC / HCC	No comparable phase II/III ctDNA-guided practice-changing trial identified in the current synthesis	—	—	This evidence gap is itself important: outside CRC, most current ctDNA data remain cohort-based, prognostic, or monitoring-oriented rather than interventional.

Note: This table is intended to separate published empirical evidence from ongoing interventional strategy trials. Where randomized ctDNA-guided trials are not available, the most clinically informative prospective cohorts or meta-analyses are shown instead. Results are presented as representative anchors and should not be interpreted as directly comparable across tumor types because assay class, sampling windows, and

endpoints differ substantially. **Abbreviations:** ACT, adjuvant chemotherapy; CRC, colorectal cancer; ctDNA, circulating tumor DNA; ddPCR, droplet digital polymerase chain reaction; DFS, disease-free survival; GEJ, gastroesophageal junction; HCC, hepatocellular carcinoma; HR, hazard ratio; mCRC, metastatic colorectal cancer; MRD/mRD, molecular residual disease; nCRT, neoadjuvant chemoradiotherapy; NPV, negative predictive value; ORR, objective response rate; OS, overall survival; PCR-NGS, polymerase chain reaction next-generation sequencing; PDAC, pancreatic ductal adenocarcinoma; PFS, progression-free survival; PPV, positive predictive value; RFS, recurrence-free survival; XELOX, capecitabine plus oxaliplatin.

References:

1. Tie, J.; Cohen, J.D.; Lahouel, K.; Lo, S.N.; Wang, Y.; Wong, R.; Shapiro, J.D.; Harris, S.J.; Khattak, A.; Burge, M.E.; et al. Circulating tumor DNA analysis guiding adjuvant therapy in stage II colon cancer. N. Engl. J. Med. 2022, 386, 2261–2272.
2. Tie, J.; Wang, Y.; Lo, S.N.; Lahouel, K.; Cohen, J.D.; Wong, R.; Shapiro, J.D.; Harris, S.J.; Khattak, A.; Burge, M.E.; et al. Circulating tumor DNA analysis guiding adjuvant therapy in stage II colon cancer: 5-year outcomes of the randomized DYNAMIC trial. Nat. Med. 2025, 31, 1509–1518.
3. Nakamura, Y.; Watanabe, J.; Akazawa, N.; Hirata, K.; Kataoka, K.; Yokota, M.; Kato, K.; Kotaka, M.; Kagawa, Y.; Yeh, K.H.; et al. ctDNA-based molecular residual disease and survival in resectable colorectal cancer. Nat Med. 2024, 30, 3272-3283.
4. Ando, K.; Hamabe, A.; Nakamura, Y.; Watanabe, J.; Hirata, K.; Kataoka, K.; Miyo, M.; Kato, K.; Akazawa, N.; Kagawa, Y.; et al. Molecular Residual Disease and Recurrence in Rectal Cancer Patients Undergoing Upfront Surgery: A Prospective Cohort Study. Ann Surg. 2026, 283, 13-21.
5. Sartore-Bianchi, A.; Pietrantonio, F.; Lonardi, S.; Mussolin, B.; Rua, F.; Crisafulli, G.; Bartolini, A.; Fenocchio, E.; Amatu, A.; Manca, P.; et al. Circulating tumor DNA to guide rechallenge with panitumumab in metastatic colorectal cancer: the phase 2 CHRONOS trial. Nat Med. 2022, 28, 1612-1618.
6. Iden, C.R.; Mustafa, S.M.; Øgaard, N.; Henriksen, T.; Jensen, S.Ø.; Ahlborn, L.B.; Egebjerg, K.; Baeksgaard, L.; Garbyal, R.S.; Nedergaard, M.K.; et al. Circulating tumor DNA predicts recurrence and survival in patients with resectable gastric and gastroesophageal junction cancer. Gastric Cancer. 2025, 28, 83-95.
7. Liu, Z.; Wang, G.; Yang, Y.; Su, Y.; Zhang, H.; Liu, J.; Cui, P.; Fan, X.; Yang, J.; Zhang, Z.; et al. ctDNA detects residual disease after neoadjuvant chemoradiotherapy and guides adjuvant therapy in esophageal squamous cell carcinoma. Cell Rep Med. 2025, 6, 102334.
8. Tatalovic, S.; Doleschal, B.; Kupferthaler, A.; Grundner, S.; Burghofer, J.; Webersinke, G.; Schwendinger, S.; Jukic, E.; Zschocke, J.; Danhel, L.; et al. Circulating Tumor DNA (ctDNA) Dynamics Predict Early Response to Treatment in Metastasized Gastroesophageal Cancer (mGEC) After 2 Weeks of Systemic Treatment. Cancers (Basel). 2024, 16, 3960.
9. Guo, M.Z.; Sachidanand, A.S.; Nguyen, T.; Patel, S.D.; Burns, W.R.; Burkhart, R.; Le, D.T.; Lafaro, K.; Bever, K.M.; Pishvaian, M.J.; et al. Postsurgical circulating tumor DNA as a prognostic biomarker for relapse of resected pancreatic ductal adenocarcinoma. J Gastrointest Surg. 2026, 30, 102359.

10. Yoo, C.; Jeong, H.; Jeong, J.H.; Kim, K.P.; Lee, S.; Ryoo, B.Y.; Hwang, D.W.; Lee, J.H.; Moon, D.B.; Kim, K.H.; et al. Circulating tumor DNA status and dynamics predict recurrence in patients with resected extrahepatic cholangiocarcinoma. J Hepatol. 2025, 82, 861-870.
11. Buonopane, I.R.; Saldanha, E.F.; de Menezes, J.S.A.; da Conceição, L.D.; Reis, C.M.P.; Leite, L.F.; Francischetto, T.; Peixoto, R.D.; Biachi de Castria, T. Circulating tumour DNA for a minimal residual disease assessment and recurrence risk in hepatocellular carcinoma: a systematic review and meta-analysis. Br J Cancer. 2026, 134, 699-706.
12. Sahin, I.H.; Lin, Y.; Yothers, G.; Lucas, P.C.; Deming, D.; George, T.J.; Kopetz, S.; Lieu, C.H.; Dasari, A. Minimal Residual Disease-Directed Adjuvant Therapy for Patients With Early-Stage Colon Cancer: CIRCULATE-US. Oncology (Williston Park). 2022, 36, 604-608.
13. NRG Oncology. NRG-GI008 / CIRCULATE-NORTH AMERICA trial description.
14. Taniguchi, H.; Nakamura, Y.; Kotani, D.; Yukami, H.; Mishima, S.; Sawada, K.; Shirasu, H.; Ebi, H.; Yamanaka, T.; Aleshin, A.; et al. CIRCULATE-Japan: Circulating tumor DNA-guided adaptive platform trials to refine adjuvant therapy for colorectal cancer. Cancer Sci. 2021, 112, 2915-2920.
15. Sato, S.; Nakamura, Y.; Oki, E.; Yoshino, T. Molecular Residual Disease-guided Adjuvant Treatment in Resected Colorectal Cancer: Focus on CIRCULATE-Japan. Clin Colorectal Cancer. 2023, 22, 53-58.
16. Zhou, J.; Huang, J.; Zhou, Z.; Fan, R.; Deng, X.; Qiu, M.; Wu, Q.; Wang, Z. Value of ctDNA in surveillance of adjuvant chemosensitivity and regimen adjustment in stage III colon cancer: a protocol for phase II multicentre randomised controlled trial (REVISE trial). BMJ Open. 2025, 15, e090394.
17. Zhou, J.; Zhang, X.; Liu, Q.; Li, Y.; Wu, G.; Fu, W.; Yao, H.; Wang, Z.; Xue, H.; Xu, T.; et al. Rationale and design of a multicentre randomised controlled trial on circulating tumour DNA-guided neoadjuvant treatment strategy for locally advanced rectal cancer (CINTS-R). BMJ Open. 2025, 15, e090765.
